# Inferring efficiency of translation initiation and elongation from ribosome profiling

**DOI:** 10.1093/nar/gkaa678

**Published:** 2020-08-21

**Authors:** Juraj Szavits-Nossan, Luca Ciandrini

**Affiliations:** SUPA, School of Physics and Astronomy, University of Edinburgh, Peter Guthrie Tait Road, Edinburgh EH9 3FD, UK; Centre de Biologie Structurale (CBS), CNRS, INSERM, Univ Montpellier, Montpellier 34090, France

## Abstract

One of the main goals of ribosome profiling is to quantify the rate of protein synthesis at the level of translation. Here, we develop a method for inferring translation elongation kinetics from ribosome profiling data using recent advances in mathematical modelling of mRNA translation. Our method distinguishes between the elongation rate intrinsic to the ribosome’s stepping cycle and the actual elongation rate that takes into account ribosome interference. This distinction allows us to quantify the extent of ribosomal collisions along the transcript and identify individual codons where ribosomal collisions are likely. When examining ribosome profiling in yeast, we observe that translation initiation and elongation are close to their optima and traffic is minimized at the beginning of the transcript to favour ribosome recruitment. However, we find many individual sites of congestion along the mRNAs where the probability of ribosome interference can reach $50\%$. Our work provides new measures of translation initiation and elongation efficiencies, emphasizing the importance of rating these two stages of translation separately.

## INTRODUCTION

Understanding the rationale behind codon usage bias and the role of synonymous codons in regulating protein synthesis are amongst the main open questions in molecular biology. Despite the fact that mRNA translation is a pivotal stage of gene expression, its sequence determinants are in fact still largely elusive (1). Recent advances in sequencing, such as ribosome profiling (2), have made it possible to probe translation dynamics at codon resolution, allowing for quantitative studies of translational efficiency.

Ribosome profiling (Ribo-seq or ribosome footprinting as it is often called), is an experimental technique delivering a snapshot of ribosome positions along all transcripts in the cell at a given condition. Its archetypal version has been developed at the end of the 1960s to study translation initiation (3,4), and has been extended in the 1980s to investigate the role of slow codons and ribosome pausing (5). Recently, Ingolia *et al.* (2) revamped this technique to exploit the next generation sequencing, and since then it is considered to be the state-of-the-art technique for studying gene expression at the level of translation.

In short, the method consists in isolating mRNA fragments (called ‘reads’) covered by a ribosome engaged in translation (∼30 nt), which are then sequenced and aligned in order to build histograms of ribosome occupancy at codon resolution on each transcript. This technique has provided an unprecedented view on translation leading to many new discoveries (6). Examples include detecting novel translation initiation sites (7), identifying actively translated open reading frames (8), quantifying the extent of stop codon readthrough (9) and elucidating the translation of long non-coding RNAs (10).

Translational activity on a given transcript is typically assessed by the number of read counts per kilobase of transcript per million mapped reads of the sample (RPKM), which takes into account the length of the transcript and the size of the sample. The RPKM is proportional to the ribosome density, which in turn is assumed to be proportional to the rate of translation—the more ribosomes on a transcript, the more efficient is protein synthesis. However, a large body of work based on mathematical modelling of ribosome dynamics suggests that the protein synthesis rate is negatively affected by increased ribosome density due to ribosome collisions (11–13). To which extent ribosome collisions can be found using ribosome profiling has been an active topic of research (14–18).

One of the goals of ribosome profiling is to understand how the elongation rate along the transcript depends on the choice of codons. Codon elongation rates are usually estimated assuming that the ribosome density at codon *i* is proportional to the ribosome’s dwell time on that particular codon (15–16,19–24); this assumption follows from the conservation of the ribosome current assuming no ribosome drop-off. Our estimate of the drop-off rate of ∼10^−3^ s^−1^ (obtained from the probability of premature termination estimated to 10^−4^ per codon (25,26) and the elongation rate of the order of magnitude of ≈10 codon/s) justifies the hypothesis. The inferred elongation rates are then checked against mRNA codon sequence features, such as codon usage bias, tRNA availability and mRNA secondary structures.

If ribosome collisions are not rare, then the elongation rates proxied by the inverse ribosome densities do not depend only on the molecular details of the elongation cycle, but also on the extent of slowing down due to ribosome traffic. The crux of the matter is that it is difficult to distinguish from the ribosome density alone whether the ribosome spent long time on a particular codon because of the long decoding time or because it had to wait for the downstream ribosome to move away. This distinction between the *actual* elongation rates that account for ribosome traffic and the *intrinsic* ones in the absence of other ribosomes has been well accounted for in the standard model for mRNA translation known as the totally asymmetric simple exclusion process (TASEP), which considers ribosomes moving along the transcript in a stochastic manner (11). Yet, very few of the existing studies use the TASEP to infer elongation rates from Ribo-seq; ones that do either do not infer the intrinsic rates (24) or use time consuming stochastic simulations to fit the Ribo-seq data (16,22).

In this work we develop an efficient method for inferring both actual and intrinsic codon-specific elongation rates from the ribosome profiling data based on the mathematical solution of the TASEP that we recently developed (12,27). Using the TASEP, we argue that the ribosome density alone is not sufficient to estimate the *absolute* elongation rates from the ribosome profiling data. Instead, our method infers elongation rates of an mRNA *relative* to the initiation rate of that transcript. Moreover, we propose new measures of translation efficiency that quantify the amount of ribosome traffic around the START codon and along the transcript. We apply our method to several Ribo-seq datasets in *Saccharomyces cerevisiae* and show evidence of local queuing *in vivo*.

## MATERIALS AND METHODS

### Ribosome profiling data

We have analysed publicly available ribosome profiling data of *S. cerevisiae* from Guydosh *et al.* (14), Pop *et al.* (20) and Weinberg *et al.* (23): NCBI GEO accession numbers GSM1279568, GSM1557447 and GSE75897 respectively. We downloaded the HDF5 files from Riboviz (https://riboviz.org) (28) and mapped to A-site positions according to Table 1 (29).

**Table 1.** A-site locations for various footprint sizes

Fragment size	Frame 0	Frame 1	Frame 2
27	15	15	18
28	15	15	18
29	15	X	18
30	15	18	18
31	15	18	18
32	X	18	18
33	18	18	18

After obtaining the A-site read density profiles, our method successfully optimized 345 of the total 346 genes from the Guydosh dataset for which the experimental ribosome density necessary for normalization was known from MacKay *et al.* (30). Analogously, the optimization was successful for 1051 out 1053 genes of the Pop dataset and for all 1589 genes in the Weinberg dataset. For the omitted genes the normalization was not possible because it resulted in ribosome density larger than 1.

### Notations

In this section we summarize the notations used in the paper. The main symbols for densities, rates and rates relative to initiation are given in Table 2. When the quantity is codon-specific we use the suffix *i* = 2, …, *L* to identify the codon number (the first codon after the START codon is at *i* = 2, the STOP codon is at *i* = *L*). Brackets { · } indicate a set of values: for instance {*a*_*i*_} is the set of all the values *a*_*i*_ for *i* = 2, …, *L*.

**Table 2.** Summary of the symbols used and their meaning

Symbol	Meaning
*L*	length of the mRNA (in codons, including START)
ℓ	length of the ribosome (in codons)
α	initiation rate [*s*^−1^]
*k*_*i*_	elongation rate [*s*^−1^] of codon *i*
*k*_*L*_ (or β)	termination rate [*s*^−1^]
{*k*_*i*_}	speed profile (elongation) of a given transcript
κ_*i*_ = *k*_*i*_/α	relative (to initiation) elongation rate at codon *i*
{κ_*i*_}	relative (to initiation) elongation profile
*r*_*i*_	experimental (normalized) density of codon *i*
{*r*_*i*_}	experimental (normalized) density profile
$r = \sum\nolimits _{i=2}^L r_i / (L-1)$	mean density of a given gene
ρ_*i*_	theoretical (normalized) density of codon *i*
$\rho _{i}^{\rm ILA}$	theoretical (normalized) density of codon *i* in the initiation-limited approximation
{ρ_*i*_}	theoretical (normalized) density profile
$\rho _i^\textrm {sim}$	simulated (normalized) density of codon *i*
$\lbrace \rho _i^\textrm {sim}\rbrace$	simulated (normalized) density profile

We emphasize that we use *normalized* densities, in units of ribosomes (A-sites) per codon. The total density is thus the averaged ribosome profile $r = \sum\nolimits _{i=2}^L r_i / (L-1)$, and the number of ribosomes translating an mRNA is *N* = *r*(*L* − 1).

### Mathematical model for mRNA translation

We model translation by a stochastic process called the TASEP introduced by MacDonald *et al.* (11,31). The TASEP describes ribosome dynamics on a discrete one-dimensional lattice representing the coding part of the mRNA molecule. Each lattice site corresponds to a codon, and ribosomes cover ℓ = 10 sites, as the ribosome footprint covers ∼30 nt or equivalently ∼10 codons. Ribosomes on the lattice are tracked according to the position of their A-site. A codon *i* that is occupied by the A-site of the ribosome is labelled by *A*_*i*_ and is otherwise labelled by ∅_*i*_.

**Figure 1.**
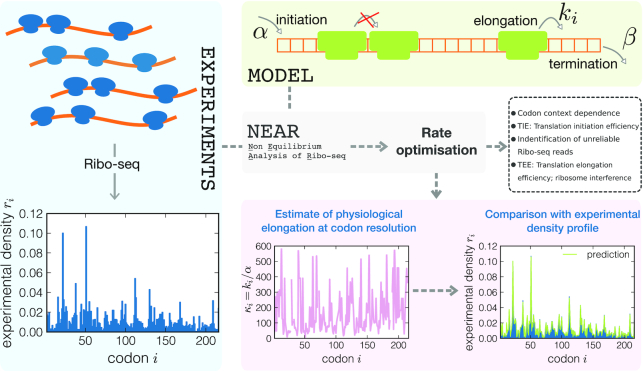
Sketch of the NEAR workflow for an individual gene (YAL007C). Experimental Ribo-seq profiles are first normalized and then analysed using the stochastic model. The normalized ribosome density profile {*r*_*i*_} is represented in the bottom left panel. The model is shown in the top right box: ribosomes covering ℓ sites are added to the lattice with an initiation rate α, provided that the first ℓ sites are not occupied by the A-site of another ribosome. Ribosomes then move from site *i* to site *i* + 1 at rate *k*_*i*_ provided that the A-site of the neighbouring ribosome downstream is at least ℓ sites away. Eventually, ribosomes leave the lattice at rate β (*k*_*L*_ = β) when their A-site is on the last site. In this drawing ℓ = 3 for clarity, whilst in our analysis we used ℓ = 10. NEAR searches for the optimal elongation rates *k*_*i*_ (relative to initiation rate α) for which the stochastic model reproduces the experimental ribosome profile. Once we find the optimal rates, we examine the extent of ribosome traffic using the translation initiation and elongation efficiencies (TIE and TEE), analyse the context dependence of elongation rates and identify problematic transcript regions in which Ribo-seq data are not consistent with the model.

A ribosome initiates translation at rate α, whereby its A-site is positioned at codon 2; this happens only if the codons 2, …, ℓ + 1 are not occupied by another ribosome’s A-site. The ribosome then advances from codon *i* to codon *i* + 1 at rate *k*_*i*_, provided that codon *i* + ℓ is not covered by the downstream ribosome (see top right drawing of the model in Figure 1). We refer to *k*_*i*_ as the intrinsic elongation rate at which the ribosome advances in the absence of other ribosomes. Eventually, when the A-site of the ribosome is at the STOP codon (the *L*-th site), the ribosome detaches the mRNA at rate *k*_*L*_ = β. Each transcript in the model is then characterized by a set of *L* rates: initiation rate α, and elongation and termination rates {*k*_*i*_} = {*k*_2_, …, *k*_*L*_}.

The process is described by the probability density *P*(*C*, *t*) to find a configuration *C* of ribosomes on an mRNA at a particular time *t*. By *configuration* we mean a particular arrangement of ribosomes described by the positions {*A*_*i*_} of their A-sites. The time evolution of *P*(*C*, *t*) is governed by the master equation:

(1) $$\begin{equation*} \frac{\textrm {d} P(C,t)}{\textrm {d} t}=\sum _{C^{\prime }\ne C}\left[W_{C^{\prime }\rightarrow C}P(C^{\prime },t)-W_{C\rightarrow C^{\prime }}P(C,t)\right], \end{equation*}$$

where $W_{C\rightarrow C^{\prime }}$ is the rate of transition from *C* to *C*′. We assume that translation takes place in the stationary limit in which case Equation (1) becomes a system of linear equations,

(2) $$\begin{equation*} 0=\sum _{C^{\prime }\ne C}\left[W_{C^{\prime }\rightarrow C}P^{*}(C^{\prime })-W_{C\rightarrow C^{\prime }}P^{*}(C)\right]. \end{equation*}$$

The three main quantities of interest are the rate of translation *J*, which measures the amount of proteins produced per unit time, the local ribosome densities ρ_*i*_, which measures the probability of detecting a ribosome at codon *i* and total ribosome density ρ, which measures the average number of ribosomes per unit length of the transcript (in codons). In the stationary TASEP, *J*, ρ_*i*_ and ρ are defined as:

(3) $$\begin{equation*} J=k_L\left\langle \tau _L\right\rangle ,\quad \rho _i=\left\langle \tau _i\right\rangle ,\quad \rho =\frac{1}{L-1}\sum _{i=2}^{L}\rho _i, \end{equation*}$$

where averaging is performed with respect to the steady-state probability P*(*C*) and τ_*i*_ is an occupation number whose value is equal to 1 if codon *i* is occupied by the A-site of the ribosome and is 0 otherwise. If we ignore premature termination due to ribosome drop-off, then *J* is constant across the transcript and is equal to the actual rate at which ribosomes initiate translation,

(4) $$\begin{equation*} J=\alpha P^{*}(\rm {first\, \ell\, codons\, free})=\alpha \left(1-\sum _{i=2}^{\ell +1}\rho _i\right), \end{equation*}$$

where P*(first *ℓ* codons free) is the steady-state probability that codons 2, …, ℓ + 1 are not occupied by an A-site.

Computing these quantities requires an exact knowledge of P*(*C*), which is known only in the biologically unrealistic case of ℓ = 1 and uniform elongation rates (32). Instead, we compute *J*, ρ_*i*_ and ρ using two approximation methods: the mean-field approximation developed in MacDonald *et al.* (11,31) and initiation-limited approximation (ILA) developed in Szavits-Nossan *et al.* (12,27). Details of these methods are presented in Supplementary Data.

### Computer program

Computer program (NEAR) for inferring elongation rates from ribosome profiling data is available under GNU General Public License v3.0 at https://github.com/jszavits/NEAR.

## RESULTS

We base our method on a well-established stochastic model for mRNA translation, the TASEP, which we describe in detail in the ‘Materials and Methods’ section. Over the years, the model has been improved in many ways to better match real translation (33,34) and has been repeatedly used to interpret experimental data (16,18,24,35–38).

In principle, the knowledge of initiation, elongation and termination rates allows one to compute simulated ribosome density profiles and protein production rates that can be compared to experimental outcomes. However, there is an open debate regarding the estimates of these rates, and no direct experimental method to measure them exists. For example, codon-specific translation elongation rates *k*_*i*_ are often assumed to be proportional to the tRNA gene copy number (GCN) or to the local tRNA adaptation index (tAI) (39–41).

Here we take a different approach and use the model to quantitatively determine codon elongation rates from ribosome profiling data. This is an *inverse problem*, since we need to optimize the inputs (parameters α and {*k*_*i*_}) in order to match the outputs (Ribo-seq data). There are three main difficulties in solving this problem, which we discuss below.

- The parameter space is extremely vast. A typical protein consists of a several hundreds of amino acids, meaning that one generally needs to optimize a comparable number of parameters.
- There is a complex non-linear relation between the set of rates {*k*_*i*_} and the ribosome density profile. A change in a single *k*_*i*_ may affect a large part of the density profile.
- Ribosome density profile predicted by the stochastic model depends only on the ratios between the elongation rates and the initiation rate, meaning that it is not possible to estimate *absolute* rates without integrating more information.

We now explain how our method tackles these problems and how it compares to existing methods that have been proposed to infer ribosome dynamics from ribosome profiling data (16,20,22,24).

Our method searches for optimal elongation rates at each codon position and separately for every transcript, i.e. we do not reduce the parameter space by assuming equal elongation rates for every instance of the same codon (20,22). Importantly, we use an analytic expression for the ribosome density profile that we recently derived in the initiation-limited regime (12,27). This relationship allows for a quick computation of the ribosome density profile instead of running costly stochastic simulations for every iteration of the optimization process (16,22). Furthermore, we emphasize that our method infers *intrinsic* elongation rates (relative to the initiation rate) related to the ribosomal elongation cycle, which may differ from the *actual* elongation rates that also take into account slowing down due to ribosome traffic (24). Thus, we are able to detect separately the mean decoding time for a particular codon and the mean time that the ribosome spends waiting for a ribosome downstream of it to move away. This distinction is central to our method.

Before we present further details of our method, we first discuss the problem of estimating absolute elongation rates, which limits the amount of information that can be inferred from ribosome profiling data alone.

### Ribosome profiles alone cannot estimate absolute elongation rates

**Figure 2.**
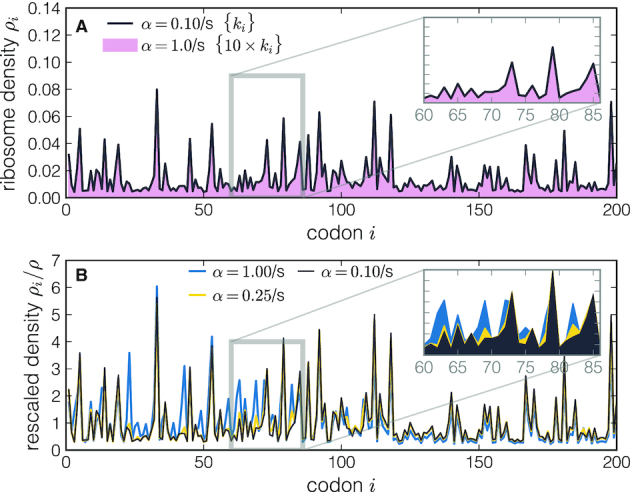
*in silico* density profiles. In panel (**A**), the black line shows the density profile obtained from the stochastic simulation of a transcript with a speed profile {*k*_*i*_} and initiation rate *α* = 0.1/s. The shadowed region corresponds to the profile of a transcript simulated with a 10-fold larger initiation rate, but keeping {κ_*i*_} constant (i.e. also increasing the elongation rates by a factor 10). This shows that densities obtained with the same elongation-to-initiation ratios {κ_*i*_} are indistinguishable. In panel (**B**), we fix the speed profile {*k*_*i*_} for three different values of the initiation rate α and we plot the rescaled profiles ρ_*i*_/ρ. As expected, by increasing the initiation rate we obtain different profiles with increasing density and traffic effects.

We remind that the ribosome density ρ_*i*_ measures the probability of detecting a ribosome’s A-site at codon position *i* (see ‘Materials and Methods’ for further details). In the Supplementary Data, we show that ρ_*i*_ depends only on the ratios between the elongation/termination rates {*k*_*j*_} and the initiation rate α–we will refer to these ratios as {κ_*j*_}. Thus given the ribosome densities {ρ_*j*_}, one can only infer the ratios {κ_*j*_}, but not the absolute rates {*k*_*j*_} and α. Since the initiation rate α is highly gene-dependent, it is not possible to compare the elongation-to-initiation ratios {κ_*j*_} from different genes without the knowledge of α for each gene. We demonstrate this point in Figure 2A, which shows the outcome of simulations of translation having different absolute rates {*k*_*i*_} but the same relative speed profile {κ_*i*_}.

We now examine two approaches that have been proposed to deal with this problem. The first approach is to fix a unique timescale shared by all mRNAs, for instance the average ribosome speed (42) or the average codon decoding time (24), which in turn allows one to estimate the initiation rate for each gene. The second approach is to normalize ρ_*i*_ by the average ribosome density ρ for that gene. This is a common practice in the analysis of Ribo-seq data, whereby the ribosome footprint read densities on individual codons are normalized by the average ribosome footprint density for that gene. The scaled read density is then assumed to be independent of the initiation rate, allowing for different genes to be compared. We argue that both of these approaches are problematic. In the first approach, the average elongation rate or the average decoding time could be highly variable from transcript to transcript, which in turn would introduce a bias when comparing absolute elongation rates between different genes. In the second approach, the normalization of ρ_*i*_ by ρ does not necessarily mean that genes with different initiation rates can be directly compared. We show that explicitly by computing ρ_*i*_/ρ in our model for different initiation rates but keeping the elongation speed profile {*k*_*i*_} fixed. As shown in Figure 2B, we find qualitatively different profiles for different initiation rates. This observation is further supported by the analytic expression for ρ_*i*_, which predicts a non-linear α-dependent correction to the linear expression ρ_*i*_ ≈ α/*k*_*i*_ (see Supplementary Data).

Instead, our approach is to scale κ_*i*_ by the termination-to-initiation ratio κ_*L*_ which removes dependence on the initiation rate since κ_*i*_/κ_*L*_ = *k*_*i*_/*k*_*L*_. Later we show that the values of κ_*L*_ inferred from ribosome profiling data in *S. cerevisiae* have amongst the least variation of all codons, which supports our choice for κ_*L*_ as the scaling factor. In addition, we introduce new measures of translation efficiency and ribosome traffic that take values between 0 and 1 and can be compared between different genes.

### Non-equilibrium analysis of Ribo-seq (NEAR)

After we have shown that the ribosome density profile alone can inform us only on the ratios {κ_*i*_} between the elongation rates and the initiation rate, we now turn to the method for inferring {κ_*i*_} from Ribo-seq. We call the method non-equilibrium analysis of Ribo-seq data (NEAR) because the model (the TASEP) that we use is borrowed from non-equilibrium statistical mechanics.

NEAR infers {κ_*i*_} with an optimization procedure that aims to find a model-predicted density profile {ρ_*i*_} which is a close match to the experimental one {*r*_*i*_} (see Figure 1). This is possible since we have recently found a mathematical expression for the ribosome density profile in terms of translation initiation, elongation and termination rates. This expression was obtained under the assumption of a limiting initiation rate α (12,27), which is supposed to hold for most of the mRNAs under physiological conditions (see Supplementary Data). However, we emphasize that the ILA does not assume that ribosome collisions are absent. Instead, our analytic solution takes ribosome collisions into account and is applicable to a wide range of initiation rates as long as they are smaller than the elongation and termination rates (see Supplementary Data).

We have applied our method to ribosome profiling data of *S. cerevisiae* obtained by Guydosh *et al.* (14), Pop *et al.* (20) and Weinberg *et al.* (23). These datasets were selected for their lack of using cycloheximide to inhibit translation elongation, which is known to distort ribosome coverage profiles (43,44). The raw data was processed by the Riboviz software (28) and mapped to A-site positions following the table provided in Ahmed *et al.* (29). After obtaining the A-site read density profiles the method proceeded in four steps, which we summarize below.

- We first normalized the number of A-site reads on each codon by the total number of reads mapped to the transcript. This number was then multiplied by the absolute ribosome density for that particular gene obtained by polysome profiling experiments in MacKay *et al.* (30). The end result is a normalized ribosome density profile {*r*_*i*_} that reveals how likely is to find a ribosome at codon *i*.
- Next, we solved a least-squares optimization problem which consisted in finding {κ_*i*_} that minimize the objective function:
  (5) $$\begin{equation*} S=\sum _{i=2}^{L}\left[{\rho _{i}^{\rm ILA}}(\lbrace \kappa _i\rbrace )-r_i\right]^2. \end{equation*}$$
  Here $\rho _{i}^{\rm ILA}$ is the model-predicted ribosome density in the ILA. The starting point for optimization were {κ_*i*_} obtained from the mean-field solution of the exclusion process. Details of $\rho _{i}^{\rm ILA}$ and the mean-field solution are presented in Supplementary Data.
- Once we found the best estimate of {κ_*i*_}, we then computed the exact density profile from stochastic simulations using the estimated {κ_*i*_}. We note that the simulated density may be different from the analytic density if the initiation rate is too high, which we checked in the next step.
- In the last step we performed two quality checks on each κ_*i*_ obtained by least-squares optimization:
  - We first verified that the ILA was applicable by comparing the analytic density with the simulated one. This step is necessary because our solution of the model is approximate and may not be valid if the initiation rate is too high, see Refs. (12,27) and also the Supplementary Data. We accepted κ_*i*_ if the relative error between the analytic and simulated density was smaller than 10 %. If not, we repeated the check using the value of κ_*i*_ obtained in the mean-field approximation.
  - For those κ_*i*_ that passed the previous check, we verified that the simulated density reproduced the experimental density *r*_*i*_ (within $5\%$ tolerance).

These are the main steps of NEAR, and we provide further mathematical details in the Supplementary Data.

We emphasize the importance of optimizing the absolute ribosome densities {ρ_*i*_} (step 2), rather than the scaled ones, {ρ_*i*_/ρ}, as in other methods that analyse ribosome profiling data (16,20). The problem is that {ρ_*i*_/ρ} remains the same if we multiply all ρ_*i*_ by a constant factor, which in turn means that different density profiles {ρ_*i*_} can result in the same scaled profile {ρ_*i*_/ρ}. Since each ρ_*i*_ is uniquely determined by the set of elongation-to-initiation ratios {κ_*i*_}, we conclude that the scaled density profile {ρ_*i*_/ρ} does not uniquely determine {κ_*i*_}, see for instance Supplementary Figure S2. Thus, we reiterate that ribosome profiling data are not sufficient to infer ribosome dynamics and in turn the extent of ribosome traffic without the additional information on the mean number of ribosomes bound per mRNA (step 1).

Our quality check in step 4 is also able to reject codons whose κ_*i*_ cannot be trusted, and identify why the inference of elongation rates for those codons is problematic. Importantly, we are able to distinguish whether our analytic solution is satisfied or not (point 4(a)), or if the problem is due to the model being inconsistent with the experimental data (point 4(b)).

Before moving on to real sequences of *S. cerevisiae*, we tested NEAR on a mock sequence with known {*k*_*i*_} (Supplementary Figure S3a), and checked that it can accurately infer the original elongation rates provided the initiation rate is not too high (Supplementary Figure S3b and c). We also remark that the quality check allows us to push the analysis to relatively high initiation rates (Supplementary Figure S3d). In those cases, however, the number of rejected codons may become significant. For a very high initiation rate we expect the ILA to fail in which case NEAR resorts to the mean-field approximation, whose estimates are further verified.

#### Using NEAR to study translation of individual genes

We demonstrate our method on a particular gene (YLR301W) using Ribo-seq data from the Weinberg dataset (23). We first compute the normalized experimental density profile {*r*_*i*_} using the experimental absolute density *r* from MacKay *et al.* (30) (in units of ribosomes/codon). This profile is then analysed following the method explained in the previous section. A set of elongation-to-initiation ratios {κ_*i*_} is obtained by optimizing the match between the model-predicted density profile and the experimental one. Each κ_*i*_ is then examined to see whether it provides a good prediction for that particular codon position and to check for inconsistencies in the method as previously explained. There are few values of κ_*i*_ that do not pass this quality check, which are rejected and are not included in the final analysis. This is a typical example, though for some genes the fraction of rejected codons is substantial and our inference procedure may be less reliable. We will come back to this point later.

**Figure 3.**
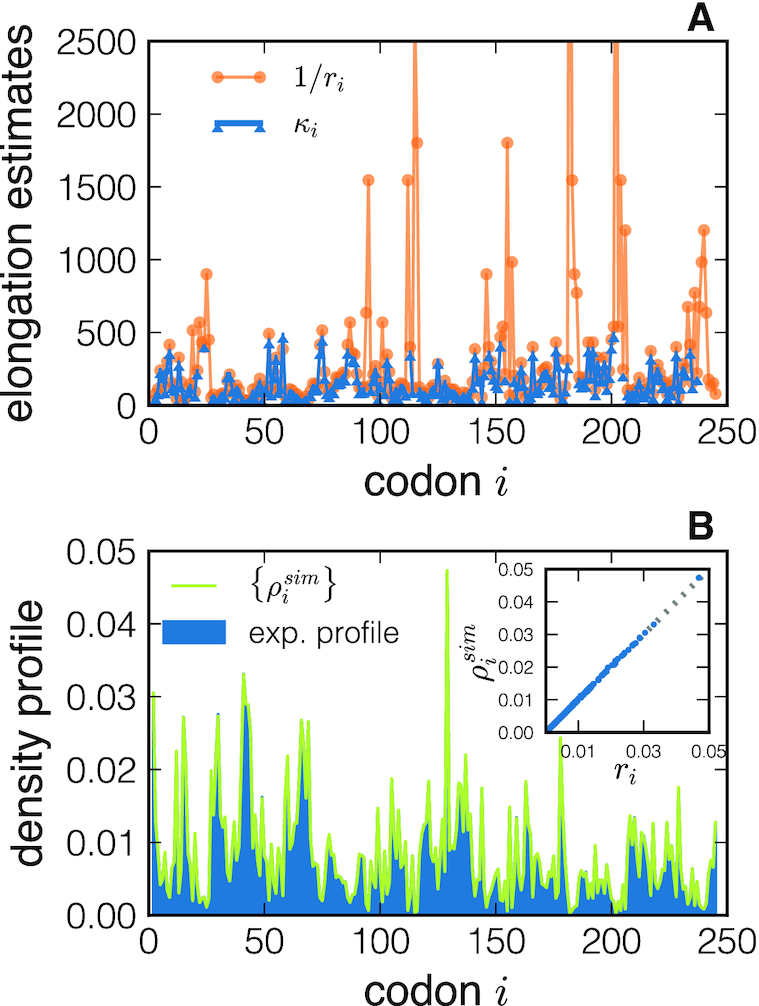
Results of NEAR applied to the YLR301W gene. (**A**) The optimized profile {κ_*i*_} is plotted (triangle markers) as a function of the codon position *i*, and compared to the naive estimates {1/*r*_*i*_} (round marker). In panel (**B**), we compare the model-predicted density profile obtained using the inferred {κ_*i*_} (lighter line) with the experimental normalized profile {*r*_*i*_}. The inset shows the scatter plot between the two densities (for each codon *i*) demonstrating an excellent agreement between theory and experiments.

In Figure 3A we plot the optimized {κ_*i*_} profile that passed the quality check (blue line, triangle markers) compared to the naive estimate 1/*r*_*i*_ (orange line, round marker) that ignores ribosome interference but it is usually judged as a good estimator of the elongation rate. We find many codon positions where the two profiles {κ_*i*_} and {1/*r*_*i*_} significantly differ from each other. Moreover, we identify values of κ_*i*_ that are not consistent with the model, whilst this cannot be done when using {1/*r*_*i*_} as a proxy for elongation determinants.

The result of a stochastic simulation of ribosome dynamics performed with the optimized elongation ratios {κ_*i*_} is shown in Figure 3B. The agreement between the simulated density profile $\lbrace \rho _i^\textrm {sim}\rbrace$ (green line) and the experimental one (in blue) is excellent for most of the codons. The inset shows the scatter plot between the values (for each codon) of the simulated and experimental ribosome density.

### Estimate of elongation-to-initiation ratios at codon resolution in yeast

We analysed three different datasets (14,20,23) and gathered the NEAR elongation-to-initiation ratios {κ_*i*_} for each gene. The percentage of codons that passed the quality check (points 4(a) and (b)) for the Weinberg, Pop and Guydosh datasets is 75, 66 and 44%, respectively. These are the percentages of the total number of analysed codons, i.e. without taking into account different transcript lengths.

We also computed the percentage of rejected codons for each transcript. The percentages of codons that were rejected at point 4(a) have a median value of 2.3% (Weinberg), 3.8% (Pop) and 8.2% (Guydosh). The respective medians for the percentages of codons that passed 4(a) but were rejected at point 4(b) are 9.5, 16 and 26.7%. Again, the best fit is achieved for the Weinberg dataset.

We note that our analysis included only transcripts with large number of reads per codon (10 or more), i.e. with high ribosome traffic. If we had analysed all transcripts, the percentage of accepted codons would have been higher. However, many transcripts with low read count have codons with zero reads, which are difficult to handle in the model as they imply unphysically large elongation speed.

**Figure 4.**
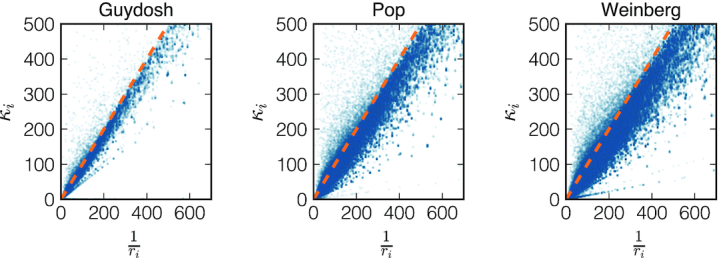
Scatter plot of the elongation-to-initiation κ_*i*_ for each codon that passed the quality check versus the inverse of the experimental density 1/*r*_*i*_ for the Guydosh, Pop and Weinberg datasets. The dashed line corresponds to the bisect.

We now turn to the codons that passed the quality check. The estimated elongation-to-initiation ratios passing the quality check are plotted in Figure 4 and compared to the naive estimates 1/*r*_*i*_. In particular, we find many instances where 1/*r*_*i*_ deviates from κ_*i*_ obtained by NEAR. The model predicts that κ_*i*_ ≈ 1/*r*_*i*_ if there are very few ribosomes on the transcript so that ribosome collisions are rare. Our findings in Figure 4 thus suggest that the effect of ribosome interference is not negligible. We will discuss this point later when we introduce better measures for detecting ribosome interference.

Next, we wanted to understand if each codon type has a characteristic decoding time and verify or reject a common hypothesis that elongation rates are determined by the availability of aminoacyl-tRNAs. By definition, κ_*i*_ is equal to the ratio *k*_*i*_/α between the elongation rate of codon *i* and the initiation rate α of the gene. Because the initiation rates are likely to be gene-specific, we do not know if the observed variation in elongation-to-initiation ratios of the same codon types (see Supplementary Figure S4) is due to variation in the elongation or initiation rates.

However, we observe that STOP codons show the least variability of all the elongation-to-initiation ratios {κ_*i*_} in the Guydosh and Weinberg datasets (Supplementary Figure S4). This result is consistent with the expectation of a context-independent termination. Thanks to this observation, we then compute the elongation-to-termination ratio κ_*i*_/κ_*L*_ = *k*_*i*_/β, i.e. the elongation rate of codon *i* with respect to the termination of the gene under investigation (Supplementary Figure S5). This quantity does not depend on the initiation rate α that is likely to be context-dependent and different from gene to gene. Indeed, the variation in κ_*i*_/κ_*L*_ linked to the same codon type is now more uniform across 61 codon types, especially in the Guydosh dataset (Supplementary Figure S5). We have also compared median values of κ_*i*_/κ_*L*_ for each codon type against two common measures of tRNA availability: a codon-dependent rate of translation based on the tRNA GCN corrected for the wobble base pairing from Weinberg *et al.* (23), and the tAI (45). We find a moderate correlation between the median of the κ_*i*_/κ_*L*_ distributions and the corresponding tRNA GCNs (Supplementary Figure S6). This result suggests that the elongation speed of individual codons is only partially determined by their codon type.

We now turn to ribosome traffic and its impact on translation efficiency. In the following sections we will define quantities that, contrary to the κ_*i*_, can be used to compare translation efficiency of different genes. Those quantities, which we name the translation initiation efficiency (TIE) and the translation elongation efficiency (TEE), can be used to rank initiation of different transcripts and quantify the impact of ribosome interference along a mRNA.

### Translation Initiation Efficiency (TIE)

By running stochastic simulations with the inferred κ_*i*_ we can measure the ribosomal current *J* divided by the initiation rate α, which is a quantity dependent on {κ_*i*_} only; the current *J* can be used as a proxy for protein synthesis rate per mRNA.

In the biological literature translation initiation is often identified with protein synthesis rate, i.e. *J* = α. However this is true only if initiation is much slower than elongation so that essentially only one ribosome is translating a transcript at a given time. Yet, this approximation is too crude to quantitatively describe translation (12). Instead, when more than one ribosome is engaged in translation, *J* becomes a function of α and the elongation rates {*k*_*i*_}; the current *J* can be thought of as the intrinsic initiation rate α multiplied by the probability that the first codons of the mRNA are not occupied by another ribosome (which would otherwise obstruct initiation).

**Figure 5.**
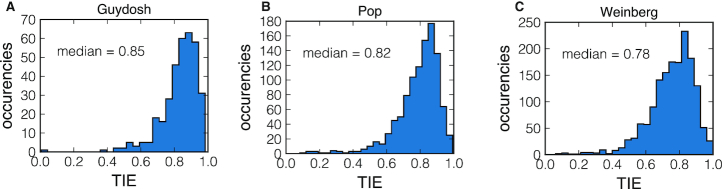
Histogram of estimated TIE for all *Saccharomyces cerevisiae* genes included in our study for the Guydosh, Pop and Weinberg datasets. The TIE gives the probability that the first codons are unoccupied.

Therefore we propose to use *J*/α as a measure of the TIE, which takes values between 0 and 1. The TIE would be equal to 1 in the optimal case in which initiation is not hindered by ribosome traffic (*J* = α, hence TIE = 1). Otherwise, the TIE gives the probability that the first codons, potentially interfering with ribosome recruitment and initiation, are unoccupied. A TIE smaller than 0.5 means that more than half of the times a new ribosome tries to initiate, it fails because of another ribosome whose A-site is located within the first 10 codons. In Figure 5 we plot the histograms of TIE for all the genes and datasets included in our study. We find that almost all genes show TIE > 0.5 with a median value around 0.8 for all the datasets. These values suggest that the first codons are mainly free from ribosomes that are already engaged in translation.

Our previous theoretical work on the exclusion process showed that if translation is rate-limited by initiation, then TIE predominately depends on the κ_*i*_ of the first ℓ ≈ 10 codons, which is the ribosome footprint on the mRNA (12). Based on that prediction, TIE > 0.5 is a strong signature that the codon sequences, and in particular the first codons of *S. cerevisiae* genes might have been selected to optimize translation initiation.

### Translation elongation efficiency (TEE) shows congestion of ribosomes *in vivo*

In contrast to the TIE, we define an efficiency index for translation elongation that identifies local ribosome interference along the transcript and not only around the initiation region. In order to do that, we emphasize that the total time *t*_*i*_(total) that a ribosome spends with its A-site on a given codon *i* can be seen as the sum of two contributions: the time *t*_*i*_(intrinsic) needed to decode this codon and incorporate the new amino acid to the growing peptide chain, plus the time *t*_*i*_(collision) spent in a queue waiting for the downstream ribosome to move. For each codon *i*, *t*_*i*_(total) = *t*_*i*_(intrinsic) + *t*_*i*_(collision). The inverse of *t*_*i*_(total) is the actual elongation rate, whilst the inverse of *t*_*i*_(intrinsic) is the intrinsic elongation rate *k*_*i*_, i.e. the elongation rate in the absence of other ribosomes. The distinction between these two rates is important because the actual elongation rates may be much smaller than the intrinsic ones in genes with higher initiation rates in which ribosomal collisions are more likely. Thus analysing the actual instead of intrinsic elongation rates could obscure our search for the molecular determinants of the translation speed.

We consider a codon as *efficient* if a ribosome attempting to translate it is not blocked by other ribosomes. We thus define the TEE at codon *i* (TEE_*i*_) as the ratio of intrinsic and total time: TEE_*i*_ = *t*_*i*_(intrinsic)/*t*_*i*_(total), or put differently, as the ratio between the actual and intrinsic elongation rate. The TEE_*i*_ is a measure of the local mRNA congestion seen by a ribosome translating the codon *i* and it depends on the context at which the codon is placed. Mathematically, it is equivalent to the probability that the *i* + 1…*i* + ℓ codons are not occupied, given that a ribosome’s A-site is at site *i*. If the intrinsic decoding time of the ribosome is equal to the total time dwelt on the codon, then the ribosome experiences on average no interference with other ribosomes and TEE_*i*_ = 1. Otherwise, 0 < TEE <1. In the extreme case of the completely jammed ribosome one would get TEE_*i*_ ≈0, i.e. the ribosome is ready to advance but it is not allowed to move forward because the transcript is overcrowded. Furthermore there is a relationship between the TEE_*i*_ and TIE given by TEE_*i*_ = TIE/(κ_*i*_ρ_*i*_). Further details are given in Supplementary Data.

We note that the TEE_*i*_ is a function of {κ_*i*_} only, meaning that ribosome interference is governed by the balance between initiation and elongation rates. A TEE profile that is close to 1 means that initiation is not frequent enough to cause ribosome congestion along the transcript. Inferring TEE profile from ribosome profiling data is thus a convenient method for testing whether translation is limited by initiation. We also stress that both the TIE and TEE_*i*_ are dimensionless quantities that take values between 0 and 1. Therefore it is possible to compare the TIE and TEE profiles between different genes.

**Figure 6.**
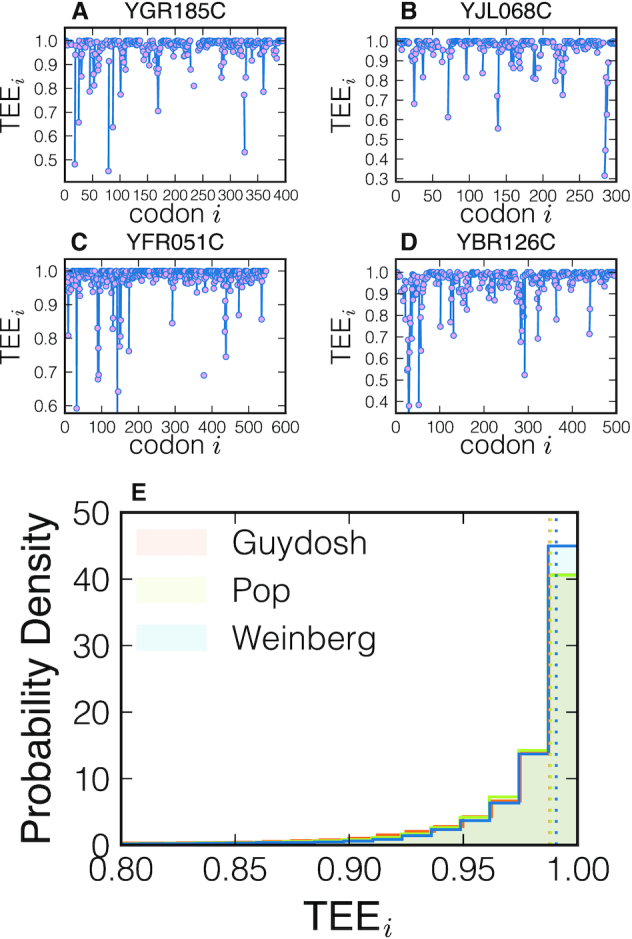
Panels (**A**–**D**) show single-gene TEE profiles (randomly selected genes from Weinberg dataset). Each point represents a codon, blue lines connects adjacent codons. Isolated points mean that their neighbouring codons have been rejected. In panel (**E**) we plot the distributions of the TEE collected on all codons of the three datasets analysed. The dashed vertical lines represent the median of those distributions (Guydosh: 0.988, Pop: 0.988, Weinberg: 0.990).

In Figure 6A–D we plot the TEE profile of four randomly selected genes. We observe that the TEE is typically close to 1 indicating that traffic is negligible for most of codons. We also identify particular codons where ribosome interference is significant and TEE_*i*_ drops to 0.6. These examples demonstrate that NEAR can locate, at codon resolution and excluding unreliable estimates, particular regions on the transcript that are affected by ribosome interference.

After analysing TEE profiles of all genes included in our study, we observe that the distribution of TEE_*i*_ for all the codons that passed the NEAR quality check is peaked at 1, with the median at about 0.99, as shown in Figure 6E. This result is consistently found in all three datasets that we analysed, suggesting that ribosome interference is present only locally on a few codons, and is generally absent *in vivo*.

**Figure 7.**
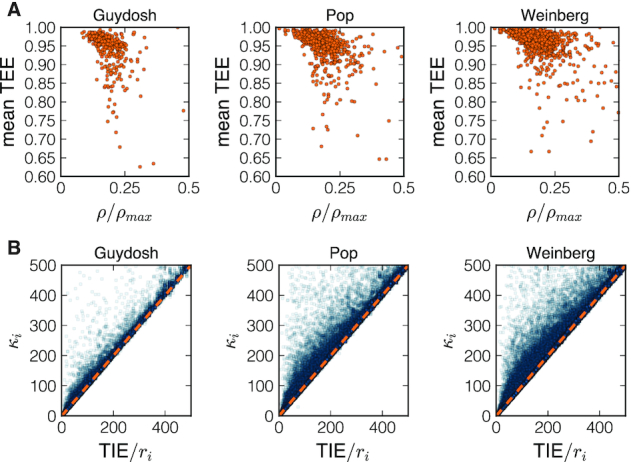
In the first row (**A**) we show the ribosome density ρ normalized with the maximal density *ρ*_max_ versus the mean value of TEE along the transcript. In the second raw (**B**) we plot *TIE*/*r*_*i*_ versus κ_*i*_ to emphasize the extent of traffic in determining the ribosome’s dwelling time. The orange dashed line is the bisect *TIE*/*r*_*i*_ = κ_*i*_ (no traffic). Codons far from that line are the ones more impacted by ribosome interference.

If the ribosome density on a given transcript is high, one would expect to see an increased number of ribosomal collisions resulting in the TEE profile that clearly deviates from 1. In Figure 7 we present the mean of the TEE profile for each gene that we analysed compared to the ratio of the ribosome density for that gene and the maximum achievable density *ρ*_max_ = 1/ℓ, where ℓ ≈ 10 codons is the ribosome footprint length. The results across all three Ribo-seq datasets clearly show that genes with low ribosome density have the mean TEE very close to 1 (few collisions). On the other hand, the mean TEE of genes with high ribosome density deviates significantly from 1 (many collisions).

Another way to demonstrate the importance of ribosome collisions is to directly estimate *t*_*i*_(collisions). Since the total time spent on a codon is given by *r*_*i*_ divided by the ribosomal current, we obtain:

(6) $$\begin{equation*} \alpha \, t_i\text{(collisions)} = \frac{r_i}{\textrm {TIE}} - \frac{1}{\kappa _i} \, . \end{equation*}$$

The time spent in traffic on codon *i* is then larger than zero if *r*_*i*_/TIE > 1/κ_*i*_ and equal to zero only if there is no ribosome interference. In the second row of Figure 7 we show that many of the codons analysed deviate from the bisect. This is another quantitative evidence that, according to experimental data, it is not that rare to observe ribosomes queuing *in vivo*.

### Initiation and elongation interdependence

**Figure 8.**
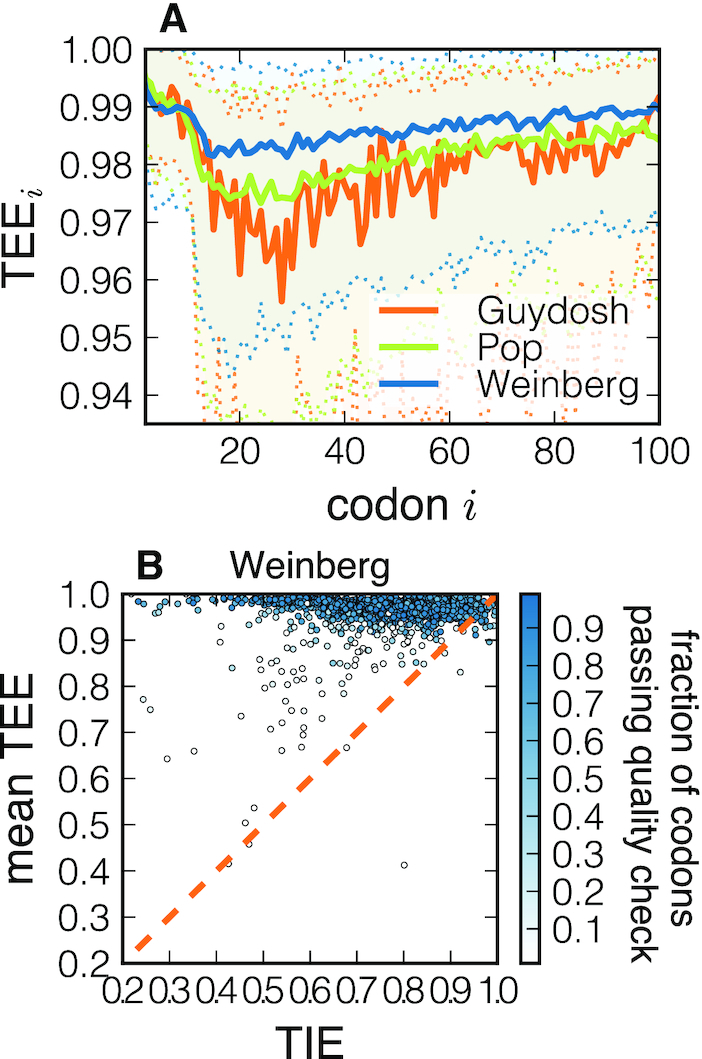
(**A**) TEE metagene profile. The full line is the median TEE profile for three different datasets included in our study. Dotted lines delimiting the shadow area correspond to the first and third quartiles of the distribution. In panel (**B**), we show the scatter plot of the TIE and mean TEE of each gene in the Weinberg dataset. The dashed line represents mean TEE = TIE. The analysis of the other datasets can be found in Supplementary Figure S8. Points are coloured according to the fraction of codons passing the quality check.

After observing that TEE is generally close to its optimum value of 1, we now look for spatial distribution of the TEE_*i*_ along the transcripts. To this end we compute a metagene TEE profile by aligning the genes at their START codon and computing the distributions of the TEE_*i*_ at each position *i*. We then take the median of the distribution on each codon. The results are plotted in Figure 8A. This genome-averaged profile confirms our earlier observation that TEE is close to 1. However, we also observe that the first ∼10 sites have a larger elongation efficiency. A large value of TEE around the START codon helps to clear this region from queueing ribosomes and thus facilitates ribosome recruitment (see also *Relationship between TIE and TEE* in Supplementary Data). This result is consistent with a large value of TIE previously observed in Figure 5, and it confirms the importance of the beginning of the coding sequence in controlling translation.

Our results seem to suggest that TIE and TEE should be strongly related. On the one hand, if translation initiation is efficient but elongation is inefficient, ribosome interference would dominate and ribosomal resources would be wasted. On the other hand, effective elongation and weak initiation would still finely tune the overall protein production without harming cellular fitness. Following these considerations, from the evolutionary point of view there should exist a constraint between the relative weights of initiation and elongation, and a situation with strong initiation and weak elongation should be avoided.

We can roughly evaluate the overall elongation efficiency as the mean of the TEE_*i*_ profile of each gene, and thus associate a couple of values (TIE, mean TEE) to each gene analysed. In Figure 8B we observe that the constraint TIE < mean TEE is satisfied for most of the genes analysed (only a few exceed the TIE = mean TEE dashed line, and for very initiation-efficient genes); thus the data analysed are consistent with the hypothesis explained above. We also notice that transcripts with inefficient initiation might also present a less optimized elongation, suggesting that initiation and elongation are interdependent.

## DISCUSSION

In this work we introduce NEAR, which is based on a well-studied model borrowed from statistical physics. The model tracks individual ribosomes engaged in translation and predicts their spatial distribution on the mRNA and the rate of protein synthesis using initiation, elongation and termination rates as input parameters. Here we do the opposite—we develop a method that infers elongation-to-initiation ratios at codon resolution directly from ribosome profiling data.

We first emphasize that Ribo-seq profiles, being an averaged snapshot of the translatome, do not contain information on the absolute timescales of the process and that thus it is possible to estimate relative rates only. These rates uniquely determine the density profile and allow us to evaluate the extent of ribosome traffic along the transcript and show a possible interplay between initiation and elongation. To this end we introduce new measures of translation efficiency that we named translation initiation and elongation efficiencies (TIE and TEE, respectively). Importantly, both TIE and TEE are dimensionless scores taking values between 0 and 1, which allows us to compare ribosome traffic between different genes.

TIE is defined as the probability that a ribosome attempting to initiate translation is not obstructed by another ribosome on the coding sequence. The distribution of TIE for the three datasets that we used in this study show that ribosomes can easily access most transcripts, with the median value of 0.8 for the probability to find the initiation region unobstructed (Figure 6). Yet, we find genes with low TIE suggesting that the first codons can exert control over protein synthesis through ribosome traffic interfering with translation initiation. These results are in line with recent experimental evidence on ribosome stalling and traffic in the initiation region (17,46–47).

Similarly, TEE_*i*_ is defined as the probability that a ribosome at codon position *i* is not blocked by another ribosome downstream of *i*. The distribution of TEE_*i*_ across all transcripts shows that TEE_*i*_ is generally close to 1 suggesting that ribosome interference is negligible for most codons (Figure 6E). However, when looking at the individual gene TEE profiles, we observe that it is not so rare to find the probability of ribosome interference as high as $50\%$ (Figure 6A–D). In accordance with these results, we find more evidence of ribosome interference (lower TEE) in genes with higher ribosome density (Figure 7). We also compute the average time *t*_*i*_(collisions) that each ribosome spends on a codon due to the blockage of downstream ribosomes. If no traffic is present then *t*_*i*_(collisions) = 0. Instead, we observe many codons for which *t*_*i*_(collisions) > 0 (Figure 7).

The fact that the value of TEE at each codon must fall between 0 and 1 allows us to agglomerate all values of TEE into a ‘metagene’ profile (Figure 8). Interestingly, the median TEE shows slightly higher values at the first 10 codons, suggesting that queuing is avoided in order to allow for efficient ribosome recruitment at the start codon. This result is consistent with a recent study in which replacing the first eight codons with their slower synonymous variants significantly reduced protein expression without affecting mRNA levels (46). Furthermore, the first codons have been recognized as critical in determining protein synthesis both theoretically (12–13,48) and experimentally (47,49–51). Beyond the first 10 codons, the metagene profile of TEE further reveals a small but noticeable drop between codons 10 and 20, followed by a slow increase between codons 20 and 100. These results are consistent with the ‘ramp hypothesis’ proposing that rare codons are more frequent at the beginning of genes in order to avoid ribosome traffic further along the transcript (52).

All together, our results indicate that translation initiation is slow compared to elongation (all κ_*i*_ = *k*_*i*_/α < 1) and the coding sequence interfering with initiation is cleared efficiently (median TIE at 0.8). We also find that translation elongation is largely optimized to avoid traffic (median TEE at 0.99), although one can locally observe high levels of ribosome interference. Interestingly, despite variations in TIE between genes (Figure 5), elongation remains consistently more efficient than initiation (mean TEE > TIE, Figure 8). It is possible that the relative role of elongation and initiation is under evolutionary pressure to allow for an efficient ribosome recruitment and to avoid ribosome interference for efficient transition from initiation to elongation (52).

Perhaps the most surprising result of our study is the variability of the inferred elongation-to-initiation ratios κ_*i*_. We can affirm that there is a correlation between common indices of codon optimality, such as the local tAI, and the estimated elongation-to-initiation ratios (see Supplementary Figure S6). However, the large variability of the estimated rates of each individual codon type implies that using those indices for protein synthesis optimization or other synthetic applications will probably not lead to the expected results. Instead, our findings indicate that codon context in the sequence is as relevant as the particular codon used, and further studies should focus on the discovery of mechanisms giving rise to the codon context dependence. For instance, mRNA secondary structures might be relevant, particularly around the initiation region (49–51,53) or the amino-acid charge at the beginning of the coding sequence (42).

Our method has detected many codons at which the model is incompatible with the ribosome profiling data, particularly for genes for which we estimated high level of ribosome interference (see for instance Supplementary Figure S7). One possibility is that our results are affected by known biases during the bioinformatic analysis (54). Another source of inconsistency between the model and the data is possibly hidden in the nature of the ribosome profiling technique. Queuing ribosomes generate large footprints (14) that are usually discarded in the experimental pipeline. Intuitively, one would expect that ribosome profiling discarding large footprints would be insensitive to ribosome interference. However, we note that the model is able to capture correlations between ribosomes that are not immediately adjacent to each other. A recent theoretical study by Scott and Szavits-Nossan (48) showed that a slow codon affects ribosome density over multiple codons, although the effect subsides with the distance from the slow codon. Indeed, NEAR finds evidence of local jamming despite the experimental bias that discards jammed ribosomes. We remark that the high TIE and TEE values at the first 10 codons could also be attributed to the nature of Ribo-seq that exclude disome footprints; a recent study by Diament *et al.* (17) in fact showed that the largest concentration of disomes in *S. cerevisiae* is at the first 10 codons.

Finally, we note that some transcripts show a significant number of rejected codons whose estimated κ_*i*_ cannot be considered reliable (see Supplementary Figure S1). In those cases the best estimate we have for κ_*i*_ is the mean-field approximation that neglects correlations between closely spaced ribosomes. Consequently, TIE and TEE may become less reliable, too. Interestingly, transcripts with many rejected codons generally display low values of TIE and mean TEE (Figure 8B). There seems to be a connection between how well the TASEP fits the ribosome profiling data and the extent of ribosome traffic that needs further investigation.

To summarize, we have developed a model-based method for inferring codon-specific elongation rates (relative to the initiation rate) from ribosome profiling data. In addition, we have proposed new measures of translation initiation and elongation efficiencies that quantify the extent of ribosome traffic *in vivo* and can be used to compare different genes and experimental conditions. We believe these new scores will complement the standard indices of translation efficiency and will contribute to the understanding of this complex biological process.

Despite the tremendous importance and potential of ribosome profiling, our work emphasizes its limitations when deciphering translation dynamics such as the lack of quantification in physical units and the lack of absolute time scales. These challenges have been recognized and steps have been made recently to combine Ribo-seq with other methods for absolute quantification such as RNA-seq with spike-ins (1) and pulsed stable isotope labelling of amino acids (18). Future developments of NEAR will include these data to obtain a more detailed view on translation dynamics. Another key question that quantitative studies using ribosome profiling should address in the future is the role of density normalization in order to better compare the outcome of different genes and of different organisms.

## Supplementary Material

gkaa678_Supplemental_Files
