# Supplementary material for: Inferring efficiency of translation initiation and elongation from ribosome profiling
Source: Nucleic Acids Res. 2020 Aug 21;48(17):9478–90. doi: 10.1093/nar/gkaa678 (PMC7515720; doi:10.1093/nar/gkaa678)
Supplement: gkaa678_Supplemental_Files [file gkaa678_supplemental_files.zip › Supplementary_Information___Inferring_efficiency_of_translation_initiation_and_elongation_from_ribosome_profiling (1).pdf]

Supplementary Information

Inferring efficiency of translation initiation and elongation from ribosome profiling

Juraj Szavits-Nossan[1,*] and Luca Ciandrini[2,+]

[1]SUPA, School of Physics and Astronomy, University of Edinburgh, Peter Guthrie Tait Road, Edinburgh EH9 3FD, United Kingdom
[2]Centre de Biologie Structurale (CBS), CNRS, INSERM, Univ Montpellier, Montpellier, France
[*]jszavits@staffmail.ed.ac.uk
[+]luca.ciandrini@umontpellier.fr

1 Analytic results in the steady state as a basis of NEAR

The model has been defined in the Materials and Methods section of the main text. Here we explain how to compute the ribosomal current J and the local ribosome density ρ_i using two approximate approaches: the mean-field approximation developed in MacDonald *et al.* [1, 2] and Shaw *et al.* [3], and initiation-limited approximation developed in Szavits-Nossan et *al* [4, 5].

The theory summarised in this Supplementary Data constitutes the basis of NEAR, whose procedure is given in the section Non-Equilibrium Analysis of Ribo-seq (NEAR) of the main text.

1.1 Ribosome density and current in the mean-field approximation

In the mean-field approximation, correlations between ribosomes are ignored, which leads to the following system of equations for J and ρ_i,

$$J = \alpha\left(1 - \sum_{s=1}^{\ell}\rho_{i+s}\right), \quad i = 1 \tag{1a}$$

$$J = k_i\rho_i \frac{1 - \sum_{s=1}^{\ell}\rho_{i+s}}{1 - \sum_{s=1}^{\ell}\rho_{i+s} + \rho_{i+\ell}}, \quad i = 2, \dots, L-\ell \tag{1b}$$

$$J = k_i\rho_i, \quad i = L-\ell+1, \dots, L. \tag{1c}$$

It is straightforward to invert these equations to get the ratio k_i/α as a function of the local densities $\rho_2, \dots, \rho_L$:

$$\frac{k_i}{\alpha} = \frac{\left(1 - \sum_{s=1}^{\ell}\rho_{1+s}\right)\left(1 - \sum_{s=1}^{\ell}\rho_{i+s} + \rho_{i+\ell}\right)}{\rho_i\left(1 - \sum_{s=1}^{\ell}\rho_{i+s}\right)}, \quad i = 2, \dots, L-\ell \tag{2a}$$

$$\frac{k_i}{\alpha} = \frac{1 - \sum_{s=1}^{\ell}\rho_{1+s}}{\rho_i}, \quad i = L-\ell+1, \dots, L. \tag{2b}$$

We will use these expressions as a starting point for the nonlinear least-squares minimisation procedure described in Section 1, which is the core of NEAR.

1.2 Ribosome density and current in the initiation-limited approximation

Recently we introduced a power-series method for computing J, ρ_i and ρ by expanding steady-state probability $P^*(C)$ in the initiation rate α,

$$P^*(C) = \sum_{n=0}^{\infty} c_n(C)\alpha^n. \tag{3}$$

The method then delineates how to compute the coefficients $c_n(C)$ recursively starting from $n = 0$. Due to its recursive nature, the power-series method is much faster than a stochastic simulation. This is absolutely crucial in order to infer $\{k_i/\alpha\}$ from $\{\rho_i\}$, which is the main part of NEAR.

It turns out that most coefficients $c_n(C)$ for small n are in fact equal to zero, which considerably simplifies the calculation of $P(C)$. Specifically, if $N(C)$ denotes the number of ribosomes in a configuration C then

$$c_n(C) = 0 \text{ if } N(C) > n. \tag{4}$$

This non-trivial result follows from a graph-theoretical interpretation of Markov chains applied to the TASEP, see Szavits-Nossan et *al.* (2018a) [4] and Szavits-Nossan et *al.* (2018a) [5] for more details. Another useful relation is

$$\sum_C c_0(C) = 1, \quad \sum_C c_n(C) = 1,\ n \geq 1, \tag{5}$$

which follows from the fact that the sum of $P(C)$ over all C is equal to 1.

If initiation is slow we can keep the first K terms in the series (3) and ignore all the rest, which we call the initiation-limited approximation (ILA),

$$P^*(C) \approx \sum_{n=0}^{K} c_n(C)\alpha^n. \tag{6}$$

In this work we compute coefficients up to and including the third order ($K = 3$), which we present in detail below. According to Eq. (4), for $K = 3$ we only need to consider configurations with at most three ribosomes on the lattice. These are $C = \emptyset$ (no ribosomes), $C = A_i$ (one ribosome at codon i), $C = A_iA_j$ (two ribosomes at codons i and j) and $C = A_iA_jA_k$ (three ribosomes at codons i, j and k).

For $n = 0$ (zeroth order), $c_0(C) = 1$ if the lattice is empty ($C = \emptyset$) and is equal to 0 otherwise, which leads to $P(C) = 1$ if we ignore higher-order terms. This is equivalent of saying that if translation initiation is not allowed ($\alpha = 0$), then the transcript will become completely empty with probability 1.

For $n = 1$ (first order), $c_1(C) \neq 0$ only if C contains at most one ribosome. The corresponding coefficients $c_1(\emptyset)$ and $c_1(A_i)$ are equal to

$$c_1(\emptyset) = -\sum_{i=2}^{L} \frac{1}{k_i}, \quad c_1(A_i) = \frac{1}{k_i},\ i = 2, \ldots, L. \tag{7}$$

These coefficients yield the following expressions for J, ρ_i and ρ in the first-order approximation

$$J = \alpha, \quad \rho_i = \frac{\alpha}{k_i}, \quad \rho = \frac{1}{L-1}\sum_{i=2}^{L} \frac{\alpha}{k_i}. \tag{8}$$

We note that the relationship $\rho_i \approx \alpha/k_i$ between ribosome densities and elongation rates that we derived above is often used in the interpretation of ribosome profiling data. Because the first order considers configurations with at most one ribosome per lattice, this result neglects the interference between ribosomes and is thus valid only for very small initiation rates.

For $n=2$ (second order), $c_2(C) \neq 0$ only if C contains at most two ribosomes. The equations for $c_2(C)$ are more involved than for $c_1(C)$ and must be solved numerically. Before we write down the equations, we introduce Kronecker delta function δ_{ij} and unit step function $\theta(i)$ defined as

$$\delta_{ij} = \begin{cases} 1 & i=j \\ 0 & i\neq j \end{cases} \quad \theta[i] = \begin{cases} 1 & i\geq 0 \\ 0 & i<0 \end{cases}. \tag{9}$$

The equations for two-particle coefficients $c_2(A_iA_j)$ then read

$$c_2(A_iA_j) = \frac{\delta_{i,2}}{e_0(A_iA_j)}c_1(A_j) + \frac{\theta[i-3]k_{i-1}}{e_0(A_iA_j)}c_2(A_{i-1}A_j) + \frac{\theta[j-1-i-\ell]k_{j-1}}{e_0(A_iA_j)}c_2(A_iA_{j-1}), \tag{10}$$

where $e_0(A_iA_j) = \theta_{j-\ell-i-1}k_i + k_j$. These equations can be solved recursively starting from $i=2$ and iterating over j from $j=\ell+2$ to $j=L$ with i held fixed. Then $i=3$ is held fixed and the iteration goes over j from $j=\ell+3$ to L and so on until $i=L-\ell$ and $j=L$. Once all $c_2(A_iA_j)$ are found, the equations for one-particle coefficients $c_2(A_i)$ are solved recursively from $i=2$ to $i=L$,

$$c_2(A_i) = \frac{\delta_{i,2}}{k_i}c_1(\emptyset) + \frac{\theta[i-3]k_{i-1}}{k_i}c_2(A_{i-1}) + \frac{\theta[L-\ell-i]k_L}{k_i}c_2(A_iA_L). \tag{11}$$

Finally, once all $c_2(A_iA_j)$ and $c_2(A_i)$ are computed, $c_2(\emptyset)$ follows from Eq. (5),

$$c_2(\emptyset) = -\sum_{i=2}^{L-\ell}\sum_{j=i+\ell}^{L} c_2(A_iA_j) - \sum_{i=2}^{L} c_2(A_i). \tag{12}$$

Using these coefficients we can compute J and ρ_i in the second-order approximation according to

$$J = \alpha - k_L\left(\sum_{j=2}^{L-\ell} c_2(A_jA_L) + c_2(A_L)\right)\alpha^2 = \alpha - \sum_{j=2}^{\ell+1}\frac{\alpha^2}{k_i} \tag{13}$$

$$\rho_i = \frac{\alpha}{k_i} + \left(\sum_{j=2}^{i-\ell} c_2(A_jA_i) + \sum_{j=i+\ell}^{L} c_2(A_iA_j) + c_2(A_i)\right)\alpha^2. \tag{14}$$

For $n=3$ (third order), $c_3(C) \neq 0$ only if C contains at most three ribosomes. The equations for three-particle coefficients $c_2(A_iA_jA_m)$ read

$$\begin{aligned} c_3(A_iA_jA_m) = {} & \frac{\delta_{i,2}}{e_0(A_iA_jA_m)}c_2(A_jA_m) + \frac{\theta[i-3]k_{i-1}}{e_0(A_iA_jA_m)}c_3(A_{i-1}A_jA_m) \\ & + \frac{\theta[j-1-i-\ell]k_{j-1}}{e_0(A_iA_jA_m)}c_3(A_iA_{j-1}A_m) + \frac{\theta[m-1-j-\ell]k_{m-1}}{e_0(A_iA_jA_m)}c_3(A_iA_jA_{m-1}), \end{aligned} \tag{15}$$

where $e_0(A_iA_jA_m)$ is given by

$$e_0(A_iA_jA_m) = \theta[j-\ell-i-1]k_i + \theta[m-\ell-j-1]k_j + k_m. \tag{16}$$

These equations are solved recursively starting from $i=2$ and $j=\ell+2$ fixed and iterating over m from $2\ell+2$ to L. Then j is increased to $\ell+3$ and the iteration over m is repeated from $2\ell+3$ to L. The procedure of increasing i and j and iterating over m is repeated so on until $i=L-2\ell$, $j=L-\ell$ and $m=L$.

Once all $c_3(A_iA_jA_m)$ are found, the equations for two-particle coefficients $c_3(A_iA_j)$ are solved recursively starting from $i=2$ and $j=\ell+2$,

$$c_3(A_iA_j)=\frac{\delta_{i,2}}{e_0(A_iA_j)}c_2(A_j)+\frac{\theta[i-3]k_{i-1}}{e_0(A_iA_j)}c_3(A_{i-1}A_j)$$
$$+\frac{\theta[j-1-i-\ell]k_{j-1}}{e_0(A_iA_j)}c_3(A_iA_{j-1})+\frac{\theta[L-\ell-j]k_L}{e_0(A_iA_j)}c_3(A_iA_jA_L). \quad (17)$$

The equations for one-particle coefficients $c_3(A_i)$ are then solved recursively from $i=2$ to $i=L$,

$$c_3(A_i)=\frac{\delta_{i,2}}{k_i}c_2(\emptyset)+\frac{\theta[i-3]k_{i-1}}{k_i}c_3(A_{i-1})+\frac{\theta[L-\ell-i]k_L}{k_i}c_3(A_iA_L). \quad (18)$$

Finally, the coefficient $c_3(\emptyset)$ is given by

$$c_3(\emptyset)=-\sum_{i=2}^{L-2\ell}\sum_{j=i+\ell}^{L-\ell}\sum_{m=j+\ell}^{L}c_3(A_iA_jA_m)-\sum_{i=2}^{L-\ell}\sum_{j=i+\ell}^{L}c_3(A_iA_j)-\sum_{i=2}^{L}c_3(A_i). \quad (19)$$

Once all coefficients for all orders up to and including the third order are computed, J and ρ_i can be computed according to the following expressions

$$J=\alpha-\sum_{j=2}^{\ell+1}\frac{1}{k_i}\alpha^2+k_L\left(\sum_{j=2}^{L-2\ell}\sum_{m=j+\ell}^{L-\ell}c_3(A_jA_mA_L)+\sum_{j=2}^{L-\ell}c_3(A_jA_L)+c_3(A_L)\right)\alpha^3, \quad (20)$$

$$\rho_i=f_i^{(1)}\alpha+f_i^{(2)}\alpha^2+f_i^{(3)}\alpha^3, \quad (21)$$

where the coefficients $f_i^{(1)}$, $f_i^{(2)}$ and $f_i^{(3)}$ depend only on $k_2,\dots,k_L$ and are given by

$$f_i^{(1)}=\frac{1}{k_i},\quad f_i^{(2)}=\sum_{j=2}^{i-\ell}c_2(A_jA_i)+\sum_{j=i+\ell}^{L}c_2(A_iA_j)+c_2(A_i),$$
$$f_i^{(3)}=\sum_{j=i+\ell}^{L-\ell}\sum_{m=j+\ell}^{L}c_3(A_iA_jA_m)+\sum_{j=2}^{i-\ell}\sum_{m=i+\ell}^{L}c_3(A_jA_iA_m)+\sum_{j=2}^{i-2\ell}\sum_{m=j+\ell}^{i-\ell}c_3(A_jA_mA_i)$$
$$+\sum_{j=2}^{i-\ell}c_3(A_jA_i)+\sum_{j=i+\ell}^{L}c_3(A_iA_j)+c_3(A_i). \quad (22)$$

From the stationary master equation in the Material and Methods section of the main text, it follows that $P(C)$ and thus ρ_i are functions of $\{\kappa_i\}=\{k_i/\alpha\}$ only. Using this fact we can absorb α^n into $f^{(n)}(C)$ and rewrite Eq. (21) as

$$\rho_i^{\text{ILA}}=g_i^{(1)}+g_i^{(2)}+g_i^{(3)},\quad g_i^{(n)}(\kappa_2,\dots,\kappa_L)=\alpha^n f_i^{(n)}(k_2\dots,k_L),\ n=1,2,3. \quad (23)$$

2 Details of the NEAR method

The method of inferring elongation-to-initiation ratios from ribosome profiling data is summarised in the Section Non-equilibrium Analysis of Ribo-seq (NEAR) of the main text. Here we provide further details of the stochastic simulations, absolute normalisation of Ribo-seq data and non-linear optimisation for computing elongation-to-initiation ratios $\{\kappa_i\}$.

2.1 Stochastic simulations

All Monte Carlo simulations were performed using the Gillespie algorithm. In the first part of the simulation we checked the total density ρ every $100 \cdot L$ updates until the percentage error between two values of the total density ρ was less than 0.1%. After that we ran the simulation for further $M = 10^4 \cdot L$ updates during which we computed the time average of ρ_i defined as

$$\rho_i^{\text{sim}} = \frac{1}{T} \sum_{n=1}^{M} \tau_i^{(n)} dt^{(n)}, \tag{24}$$

where $\tau_i^{(n)}$ is the value of τ_i (1 if codon i is occupied by the ribosome's A-site and 0 otherwise) just before the n-th update in the simulation, $dt^{(n)}$ is the time interval between the $(n-1)$-th and the n-th iteration of the Gillespie algorithm, and $T = \sum_{n=1}^{M} dt^{(n)}$ is the total time.

2.2 Normalisation of Ribo-seq data

Genes from the three datasets analysed were selected to have at least 10 “reads" per codon on average. For each gene we computed the value of local experimental ribosome density r_i at codons $i = 2, \ldots, L$ according to

$$r_i = \rho \frac{N_i}{\sum_{n=2}^{L} N_n}, \quad i = 2, \ldots, L \tag{25}$$

where N_i is the number of reads at codon i obtained from ribosome profiling experiments, ρ is the total ribosome density and L is the number of codons for that particular gene. Most genes in our analysis had codons with zero reads, which implies an infinite value of the elongation rate. In order to mitigate this problem we replaced $N_i = 0$ by $N_i = 1$ and calculated r_i according to Eq. (25). The normalisation procedure failed for only three genes across all three datasets, because the normalised ribosome density r_i was larger than 1 at some codons.

2.3 Non-linear optimisation for computing elongation-to-initiation ratios

We looked for $\kappa_i = k_i/\alpha$ such that the theoretical ribosome density ρ_i^{ILA} predicted from the initiation-limited approximation in Eq. (21) matched the experimental density r_i,

$$\rho_i^{\text{ILA}}(\kappa_2, \ldots, \kappa_L) = r_i, \quad i = 2, \ldots, L. \tag{26}$$

The procedure of finding κ_i consisted of four steps:

1. First we estimated the values of κ_i^{MF} in the mean-field approximation according to Eq. (2).

2. Next we computed local ribosome densities $\rho_i^{\text{sim}}(\{\kappa_i^{\text{MF}}\})$ by running a stochastic simulation of the TASEP using the Gillespie algorithm in which the elongation rate k_i at codon i was set to κ_i^{MF} estimated in the mean-field approximation (i.e. setting $\alpha = 1$ in the simulation without loss of generality).

3. We then compared $\rho_i^{\text{sim}}(\{\kappa_i^{\text{MF}}\})$ to r_i for each $i = 2, \ldots, L$ and identified codons $x(1), \ldots, x(N)$ for which the percentage error between $\rho_i^{\text{sim}}(\{\kappa_i^{\text{MF}}\})$ and r_i was larger than 5%.

4. Finally, we solved a nonlinear least squares optimisation problem using $\{\kappa_i^{\rm MF}\}$ as a starting point, which consisted of finding $\kappa_{x(1)},\ldots,\kappa_{x(N)}$ for which the sum of squares

$$S(\kappa_2,\ldots,\kappa_L) = \sum_{j=2}^{L}\left(\rho_j^{\rm ILA}(\kappa_2,\ldots,\kappa_L) - r_j\right)^2 \tag{27}$$

was minimal, where $\kappa_i = \kappa_i^{\rm MF}$ for codons $i \neq x(1),\ldots,x(N)$ that were not selected for optimisation and $\rho_i^{\rm ILA}$ is given in Eq. (23).

The nonlinear optimisation was preformed using NLopt library from Ref. [6]. We used a local derivative-free algorithm called BOBYQA which was developed in Ref. [7]. In order to prevent unrealistic values of κ_i and to speed up the optimisation procedure, we restricted the search to $10^{-3} \leq \kappa_{x(1)},\ldots,\kappa_{x(N)} \leq 10^{6}$. The optimisation search was preformed until one of the following two stopping criteria was met: (1) the fractional error $\Delta S/S$ between two iterations was less than 10^{-8} and (2) the total run time exceeded 60 minutes. These numbers were chosen due to time constraints when analysing many genes; a better accuracy may be achieved for individual genes by amending the stopping criteria. After the optimisation procedure finished we recomputed local densities $\rho_i^{\rm sim}$ by running a stochastic simulation of the TASEP with elongation rates $k_i = \kappa_i$ and compared them to experimental values r_i.

2.4 Quality check of the estimated elongation-to-initiation ratios

In this section we detail the quality check that we carried out for each value of the inferred $\kappa_i = k_i/\alpha$. For each gene for which the normalisation of Ribo-seq data was successful we preformed the following seven checks:

1. *Improvement over the initial (mean-field) prediction.* We check whether the optimisation procedure has improved the agreement with respect to the initial (mean-field) prediction. For the mean-field prediction we compute the sum of squares $S_{\rm MF}$

$$S_{\rm MF} = \sum_{i=2}^{L}\left(\rho_i^{\rm sim}(\{\kappa_i^{\rm MF}\}) - r_i\right)^2, \tag{28}$$

where $\{\rho_i^{\rm sim}(\{\kappa_i^{\rm MF}\})\}$ is the simulated density profile computed with the mean-field rates $\{\kappa_i^{\rm MF}\}$ and r_i is the experimental density profile. The value of $S_{\rm MF}$ is then compared to $S_{\rm opt}$ obtained from

$$S_{\rm opt} = \sum_{i=2}^{L}\left(\rho_i^{\rm sim}(\{\kappa_i\}) - r_i\right)^2, \tag{29}$$

where $\{\rho_i^{\rm sim}(\{\kappa_i\})\}$ is the simulated density profile computed using the inferred elongation-to-initiation ratios $\{\kappa_i\}$. If $S_{\rm opt} < S_{\rm MF}$ then the TIE is taken from the simulations of the optimised system, otherwise from the MF simulations.

2. *Rate-limiting step in translation.* We verify if the optimised elongation-to-initiation ratio $\kappa_i > 1$ $\forall i$ (otherwise initiation is not the limiting step and the initiation-limited approximation cannot be used).

3. *Applicability of the initiation-limited approximation.* We set a tolerance ε_{ILA} for the ribosome density ρ_i^{ILA} predicted by the initiation-limited approximation in Eq. (21). For each codon we check if $|\rho_i^{\text{ILA}} - \rho_i^{\text{sim}}(\{\kappa_i\})|/\rho_i^{\text{sim}} < \varepsilon_{\text{ILA}}$. If that is the case the power series approximation holds and the method is reliable. We set $\varepsilon_{\text{ILA}} = 0.1$.

4. *Comparison with Ribo-seq data.* If the codon passes the quality checks in points 2 and 3 then we check if the prediction is consistent with the experimental profile $\{r_i\}$ (within a tolerance ε_{EXP}) by checking if $|r_i - \rho_i^{\text{sim}}(\{\kappa_i\})|/r_i < \varepsilon_{\text{EXP}}$, where we set $\varepsilon_{\text{EXP}} = 0.05$. Codons that pass this check are kept and considered reliable only if $\kappa_i < \kappa_{\text{thr}}$. This last check is to discard values of κ_i that are deemed suspiciously large and are likely unrealistic. Such unreasonably large values of κ_i are due to low number of reads for a particular codon, especially for codons for which we artificially increased the number reads from zero to one in order to avoid infinite elongation rates. Because such small number of reads may be due to experimental errors, we decided to exclude those codons from the final analysis. We used the threshold value $\kappa_{\text{thr}} = 500$ corresponding to an elongation rate k= 60/s (assuming $\alpha = 0.12$/s).

5. *Problematic codons.* If $|r_i - \rho_i^{\text{sim}}(\{\kappa_i\})|/r_i > \varepsilon_{\text{EXP}}$ then the codon is excluded from the final analysis and we set $\kappa_i = -1$ (to identify the problematic codon for further analysis). Those codons fall in the most interesting class, in which the experimental data cannot be reproduced using the existing theory. We speculate that those "problematic" codons might also arise because ribosome profiling cannot detect clusters of ribosomes that will instead be predicted by our simulated profile $\{\rho_i^{\text{sim}}(\{\kappa_i\})\}$.

   The fraction of problematic codons for each of the three datasets is presented in Figure S1. A full list of codons entering in this category (in which our method can be applied but experimental data are inconsistent with the density generated by NEAR) is in the Supplementary Files 1,2,3.

6. *Falling back to the mean-field prediction.* In the case in which the codon does not pass the quality checks in points 2 and 3, the mean-field prediction $\{\kappa_i^{\text{MF}}\}$ will be considered. If $|r_i - \{\rho_i^{\text{sim}}(\{\kappa_i^{\text{MF}}\})\}|/r_i < \varepsilon_{\text{EXP}}$, then κ_i^{MF} is kept and the mean-field prediction is considered reliable. Otherwise the codon is excluded from the final analysis and we set $\kappa_i = -2$.

7. *Stop codon.* We further check if the elongation-to-initiation ratio κ_L of the stop codon has been kept for the analysis. If κ_L is reliable then the ratio $\kappa_i/\kappa_L = k_i/k_L$ can be computed.

2.5 Computing TEE profiles

We remind the following relation from the main text: $t_i(\text{total}) = t_i(\text{intrinsic}) + t_i(\text{collision})$. By definition of TEE_i,

$$\frac{1}{t_i(\text{total})} = \frac{1}{t_i(\text{intrinsic})}\,\text{TEE}_i\,. \tag{30}$$

The ribosomal current is given by

$$J = k_i \rho_i P(\ell \text{ free codons } |i)\,, \tag{31}$$

where $P(\ell \text{ free codons } |i)$ is the probability that the $i+1 \dots i+\ell$ codons are not occupied (given that a ribosome's A-site is at site i). If we identify the elongation rate k_i with $1/t_i(\text{intrinsic})$ and J/ρ_i with the effective elongation rate $\frac{1}{t_i(\text{total})}$, then by comparing Eqs. (30) and (31) we find that the TEE_i is equivalent

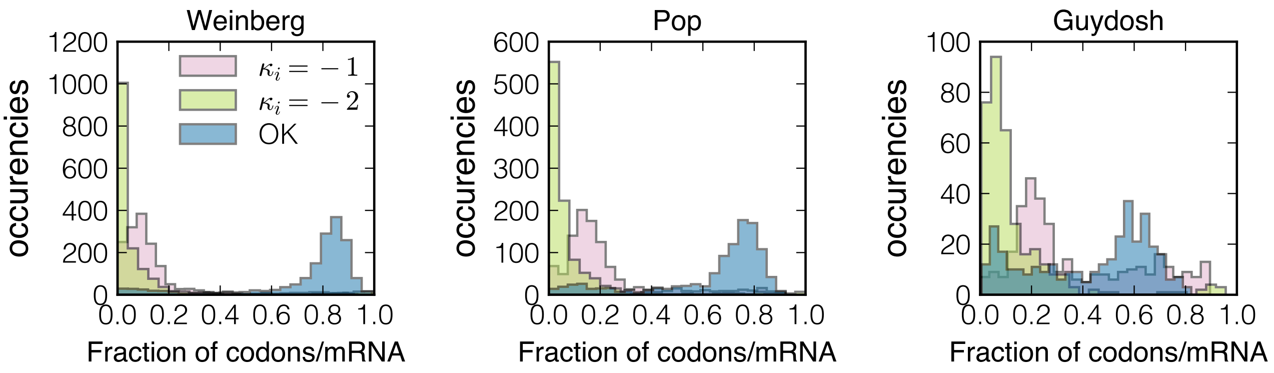

Figure S1. In pink we show the distribution of the fraction of codons per mRNA which are considered problematic (step 5, or $\kappa_i = -1$), while in green we represent the distribution of the fraction of codons per transcript that do not pass step 6 (i.e. for which the initiation-limited approximation is not valid and the mean-field is not considered reliable neither, $\kappa_i = -2$). The equivalent distribution for the fraction of codons passing the quality check is in blue. We remind that the number of analysed genes in the Weinberg, Pop and Guydosh datasets are 1589, 1051 and 345 respectively. The median fraction of codons/mRNA passing the quality check is 0.83, 0.73, 0.51 for these datasets.

to $P(\ell \text{ free codons } |i)$. Based on the definition of the TIE=J/α, we obtain the TEE profile by computing $\text{TEE}_i = \text{TIE}/(\kappa_i \rho_i^{\text{sim}})$ at each codon i at which κ_i passed the quality check. Therefore the profile is "broken" when NEAR cannot find a reliable estimate for the elongation-to-initiation ratio κ_i. We note that by definition TEE_i is a probability and as such must take values between 0 and 1. Because each of TIE, κ_i and ρ_i^{sim} comes with its own statistical error, TEE may occasionally be larger than 1. In those cases we capped the value to 1.

2.6 Relationship between TIE and TEE

Translation initiation index measures the unoccupancy of the first $\ell = 10$ codons and is thus related to κ_i and TEE_i in that region. The TIE is defined as J/α, where J is the ribosome current i.e. the number of successful translation initiations per unit time. The ribosome current is given by $J = \alpha P(\text{first } \ell \text{ codons free})$, where $P(\text{first } \ell \text{ codons free})$ is the probability that the first $\ell = 10$ codons are free. The latter is given by

$$P(\text{first } \ell \text{ codons free}) = 1 - \sum_{i=2}^{\ell+1} \rho_i, \tag{32}$$

From there we conclude that

$$\text{TIE} = P(\text{first}\ell\text{codons free}) = 1 - \sum_{i=2}^{\ell+1} \rho_i = 1 - \sum_{i=2}^{\ell+1} \frac{\text{TIE}}{\kappa_i \text{TEE}_i}, \tag{33}$$

where in the last step we used that $\rho_i = \text{TIE}/(\kappa_i \text{TEE}_i)$. Finally, the relationship between TIE, κ_i and TEE_i of the first ℓ codons is given by

$$\text{TIE} = \frac{1}{1 + \sum_{i=2}^{\ell+1} \frac{1}{\kappa_i \text{TEE}_i}}. \tag{34}$$

This expression reveals that efficient translation initiation requires both fast elongation ($\kappa_i > 1$) and no ribosome queuing (TEE_i close to 1) within the first $\ell = 10$ codons.

References

1. MacDonald,C.T., Gibbs,J.H., and Pipkin,A.C. (1968) Kinetics of biopolymerization on nucleic acid templates. *Biopolymers,* **6**(1), 1–25.
2. MacDonald,C.T. and Gibbs,J.H. (1969) Concerning the kinetics of polypeptide synthesis on polyribosomes. *Biopolymers,* **7**(5), 707–725.
3. Shaw,L.B., Sethna,J.P., and Lee,K.H. (2004) Mean-field approaches to the totally asymmetric exclusion process with quenched disorder and large particles. *Physical Review E - Statistical, Nonlinear, and Soft Matter Physics,*.
4. Szavits-Nossan,J., Ciandrini,L., and Romano,M.C. (2018) Deciphering mRNA Sequence Determinants of Protein Production Rate. *Phys. Rev. Lett.,* **120**, 128101.
5. Szavits-Nossan,J., Romano,M.C., and Ciandrini,L. (2018) Power series solution of the inhomogeneous exclusion process. *Physical Review E,* **97**(5), 1–13.
6. Johnson,S.G. The nlopt nonlinear-optimization package.
7. Powel,M.J. (2009) The bobyqa algorithm for bound constrained optimization without derivatives.Technical Report.
8. Dao Duc,K. and Song,Y.S. (2018) The impact of ribosomal interference, codon usage, and exit tunnel interactions on translation elongation rate variation. *PLoS Genetics,* **14**(1), 1–32.
9. Ciandrini,L., Stansfield,I., and Romano,M.C. (2013) Ribosome Traffic on mRNAs Maps to Gene Ontology: Genome-wide Quantification of Translation Initiation Rates and Polysome Size Regulation. *PLoS Comput. Biol.,* **9**(1).
10. Weinberg,D.E., Shah,P., Eichhorn,S.W., Hussmann,J.A., Plotkin,J.B., and Bartel,D.P. (2016) Improved Ribosome-Footprint and mRNA Measurements Provide Insights into Dynamics and Regulation of Yeast Translation. *Cell Reports,* **14**(7), 1787–1799.
11. Reis,M.d., Savva,R., and Wernisch,L. (2004) Solving the riddle of codon usage preferences: a test for translational selection. *Nucleic Acids Research,* **32**(17), 5036–5044.

3 Supplementary Figures

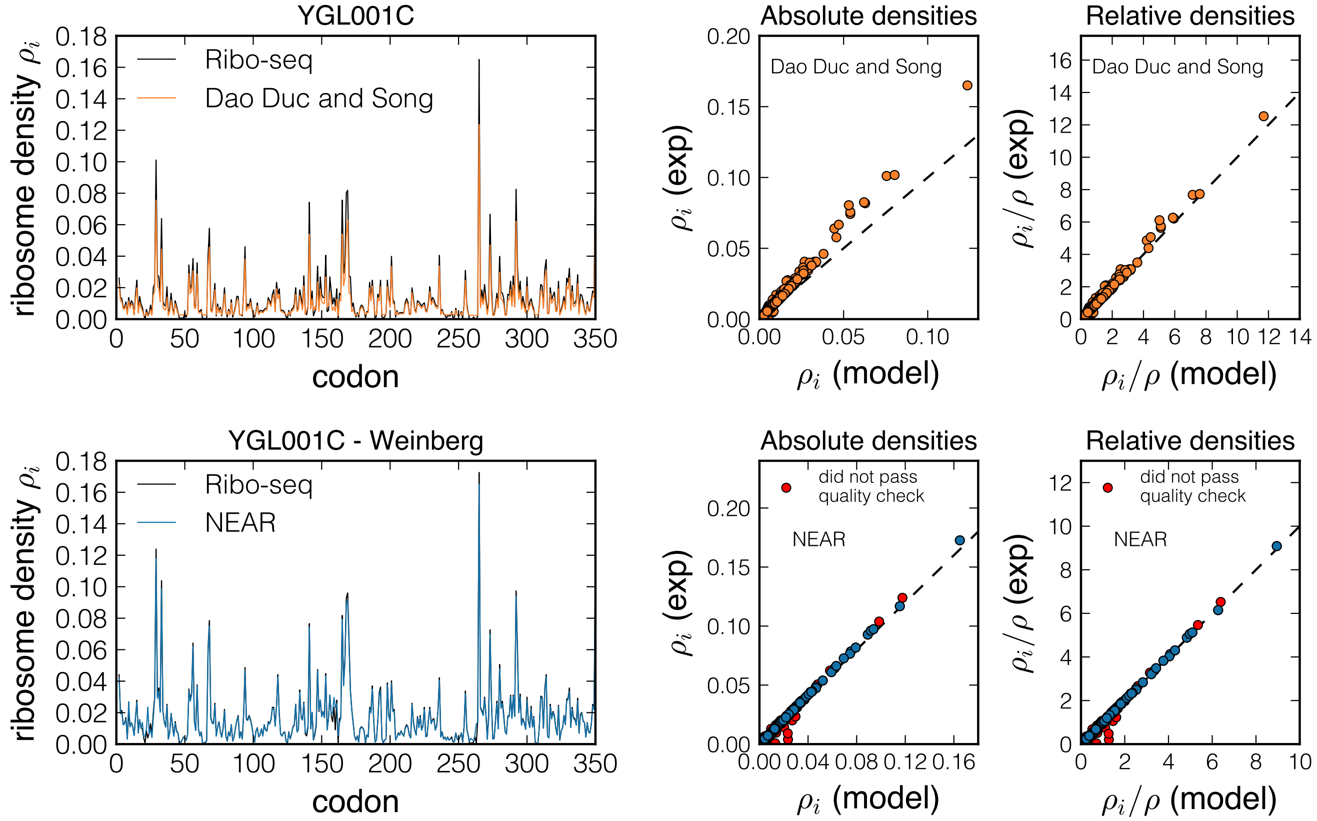

Figure S2. We compare results from Dao Duc and Song [8] (first row) and NEAR (second row). Although the density profile from simulations with rates obtained by optimising the relative density profile $\{\rho_i/\rho\}$ looks comparable with the experimental one (top-left panel), there is a bias between absolute densities (top-middle panel). This follows from the fact that $\{\rho_i/\rho\}$ remains unchanged if we multiply all ρ_i by a constant factor, which in turn means different densities ρ_i and therefore different κ_i. In the bottom-left panel, ribosome density for the same gene predicted by NEAR (blue line) is compared to the experimental data (black line). NEAR is founded on the optimisation of *absolute* densities, thus both absolute and relative densities match the experimental data (bottom-middle and right panel). In the top row we computed absolute densities as $TE/(100 \times 0.83) \times L \times R_i/R$, where TE is the translation efficiency, L is the length of the mRNA in codons, R_i is the number of reads for codon i and R is the total number of reads of that gene; data from Dao Duc and Song [8]. In the bottom row the experimental densities are the r_i introduced in the main text. We highlight in red (bottom-middle and right panel), the codons that did not pass the quality check of our method.

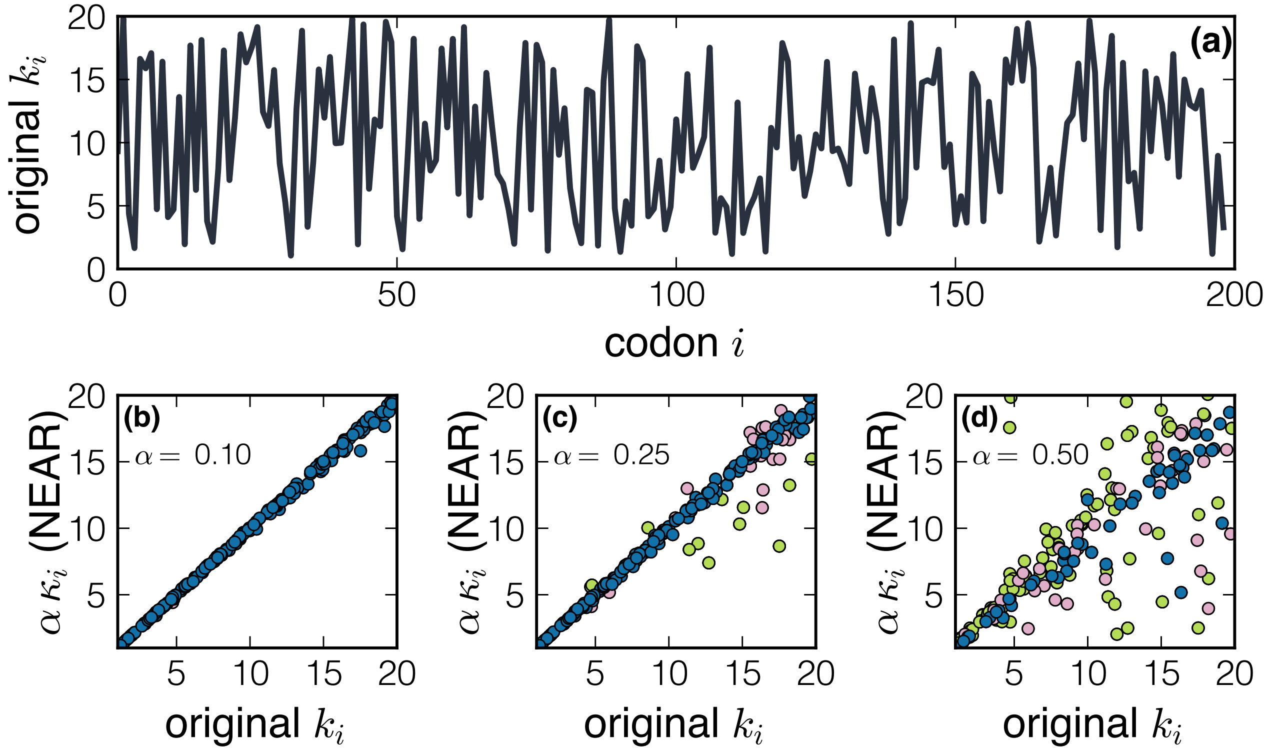

Figure S3. Testing NEAR on a random sequence of rates $\{k_i\}$. The $\{k_i\}$ profile is shown in panel (a). Panels (b)-(c)-(d) show the scatter plot between the rates $\alpha\,\kappa_i$ estimated by NEAR (y-axis) and the original rates $\{k_i\}$ (x-axis) with increasing values of the initiation rate α. The points in green are the κ_i values that did not pass the point 4(a) of the quality check as described in the main text (i.e. for which κ_i is fixed to -2 in the algorithm. Pink points are the ones that did not pass point 4(b), and for which κ_i is arbitrarily fixed to be -1. Since NEAR is based on a power series approximation that is reliable for small initiation rates, the method is expected to perform badly for large values of α. However, even in this regime the quality check is able to exclude codons that will not be considered in the final analysis. We remind that initiation has been estimated to be limiting and the physiological situation is the one presented in panel (b) for most of the genes [9]. For instance in panel (d) one would measure values of κ_i as small as ~ 2.5, while the values of κ_i estimated from datasets are generally at least on order of magnitude larger (see Figure S4).

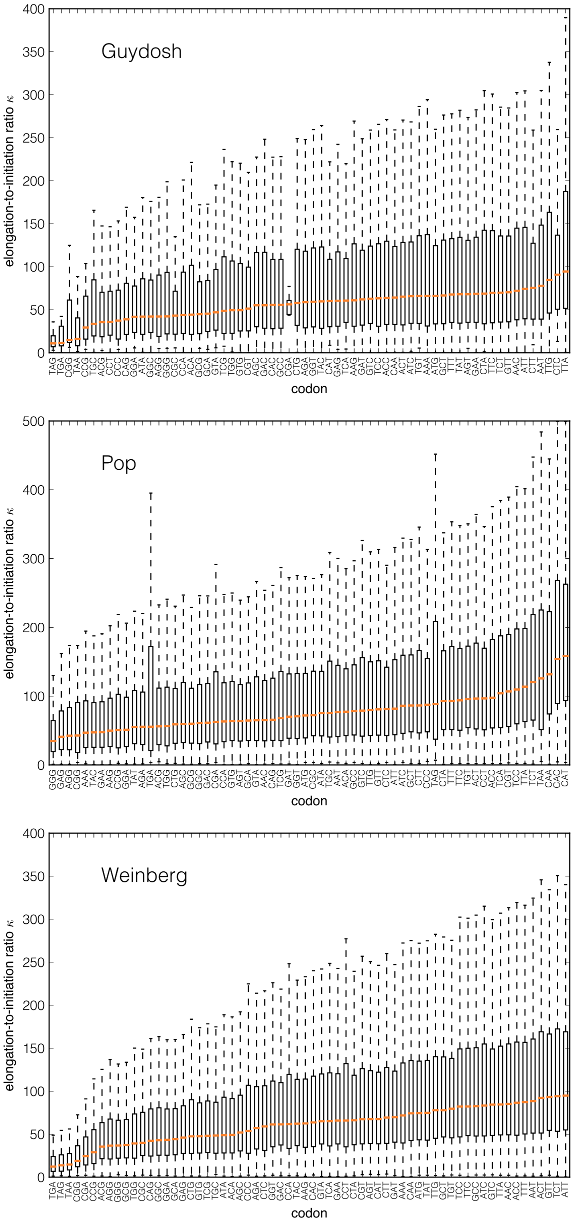

Figure S4. Distributions of elongation-to-initiation ratios κ for all codon types. We gather the ratios $\kappa_i = k_i/\alpha$ for each codon-type, and plot their distributions. In principle codons from different genes cannot be compared because they have a different initiation rate α. However, we notice a small variability in the estimates of STOP codons (TAA, TAG, TAA) for the Guydosh and Weinberg datasets. This is reasonable since STOP codons are supposed to be less context dependent. We also remark that, as opposed to what is generally believed, STOP codons are the slowest, but still ~ 10 times faster compared to initiation. The boxplots show the quartiles $Q1$ and $Q3$ ($\Delta Q = Q3 - Q1$) of the distribution, the median is the orange horizontal line; the whiskers extend to the most extreme, non-outlier data points (i.e. to the last data point less than $Q3 + 1.5\Delta Q$ and greater than $Q1 - 1.5\Delta Q$). The Guydosh dataset is the smallest one, and only 10 CGA codons passed the quality check.

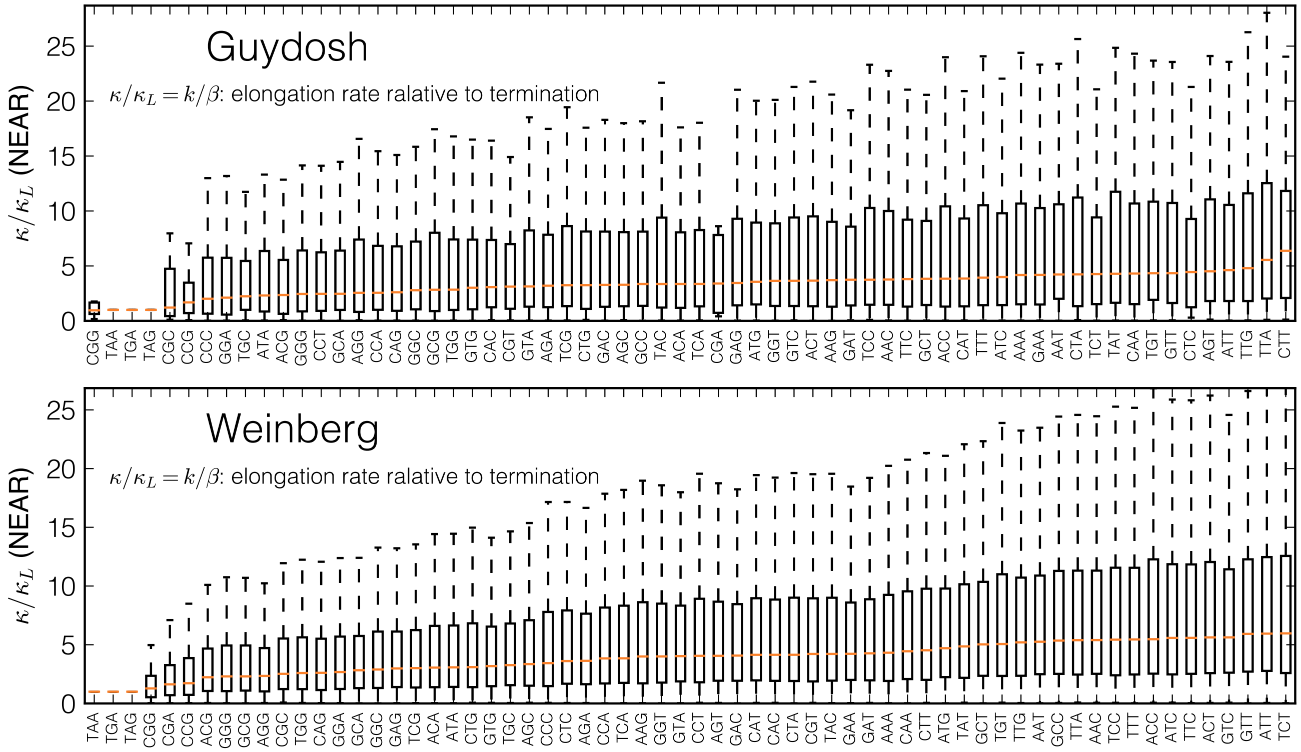

Figure S5. Boxplot of the distribution of the elongation-to-termination ratios κ/κ_L for each codon type for the Guydosh and Weinberg dataset. There is a $\approx$5-fold difference in the median value of κ/κ_L between the slowest and the fastest codon, in accordance with a common view that translation elongation is a non-uniform process. However, in we also find a large variability in the values of κ/κ_L belonging to the same codon type. This variability suggests that the elongation speed of individual codons is only partially determined by their codon type. The boxplots show the quartiles $Q1$ and $Q3$ ($\Delta Q = Q3 - Q1$) of the distribution, the median is the orange horizontal line; the whiskers extend to the most extreme, non-outlier data points (i.e. to the last data point less than $Q3 + 1.5\Delta Q$ and greater than $Q1 - 1.5\Delta Q$). Only 11 CGG codons of the Guydosh dataset can be used to plot these distributions (both CGG and STOP codon passed the quality check).

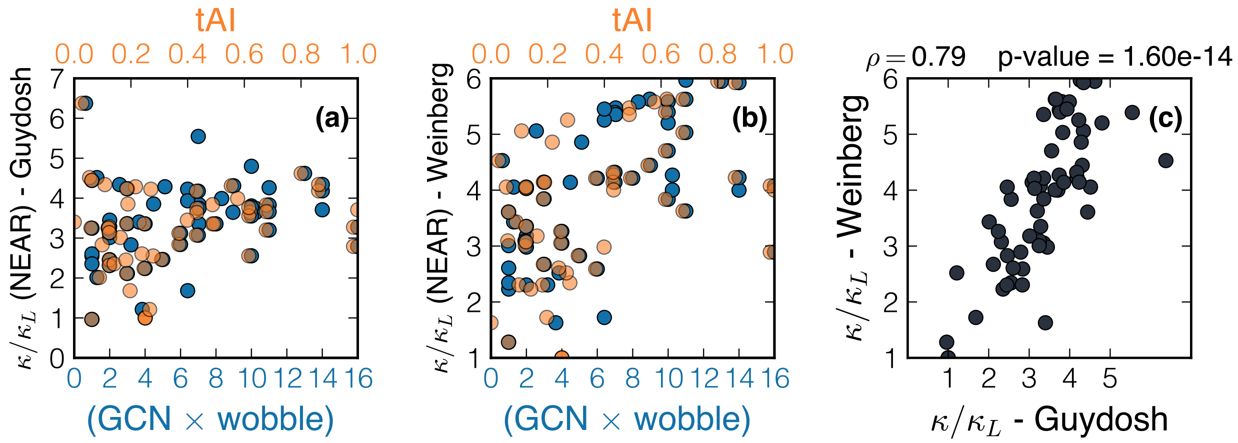

Figure S6. We compare median values of κ/κ_L against two common measures of tRNA availability, a codon-dependent rate of translation based on the tRNA gene copy number (GCN) corrected for the wobble base pairing from Weinberg *et al.* [10], and the tRNA adaptation index (tAI) [11] for Guydosh (a) and Weinberg (b) datasets. We find a moderate correlation between the median of the κ/κ_L distributions and both measures of tRNA availability (GCN × wobble: Spearman $\rho = 0.34$, $p = 0.008$ - Guydosh; $\rho = 0.57$, $p = 1.4 \times 10^{-6}$ - Weinberg; tAI: $\rho = 0.22$, $p = 0.1$ - Guydosh; $\rho = 0.46$, $p = 3 \times 10^{-4}$); this suggests that the elongation speed of individual codons is only partially determined by their codon type. Panel (c) shows the comparison between the two datasets.

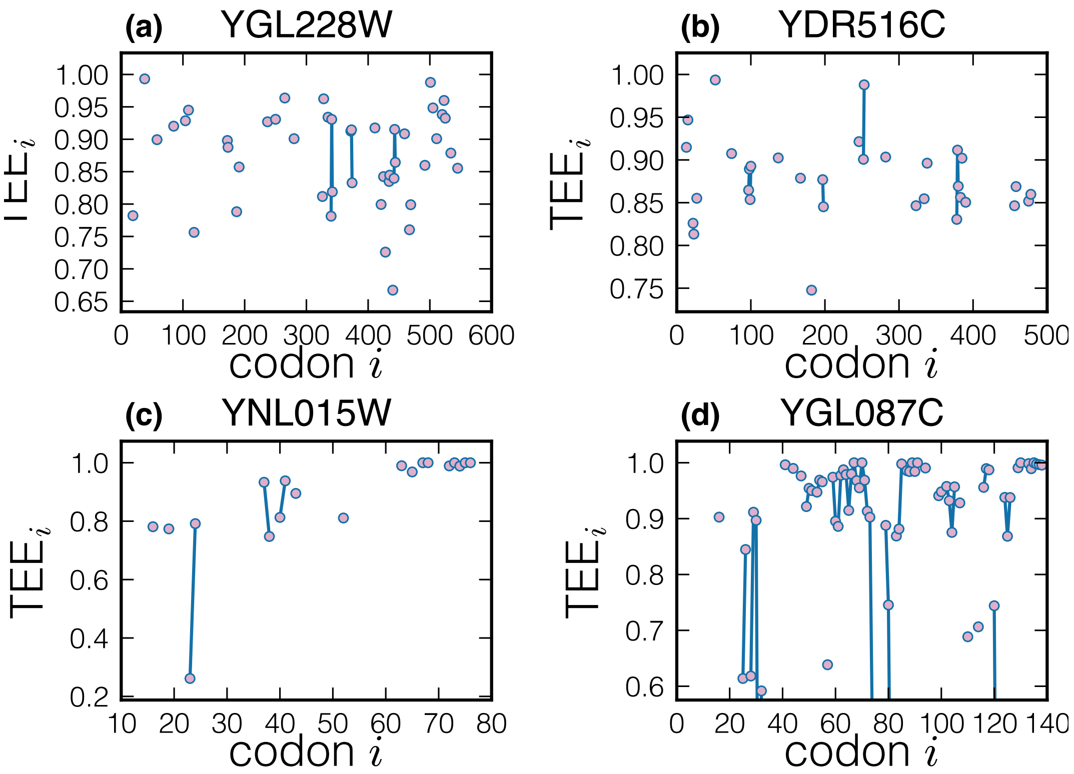

Figure S7. Profiles of TEE for four genes amongst the ones with the smallest average TEE. When the TEE is low, many κ_i do not pass the quality check, as it can be seen from the many points missing in the TEE profiles. This means that the experimental read counts are inconsistent with the model. We speculate that this is due to the bias in the ribosome profiling neglecting clusters of ribosomes. However, NEAR fills the partial information that is enclosed in the experimental profile and finds evidence of traffic (small TEE).

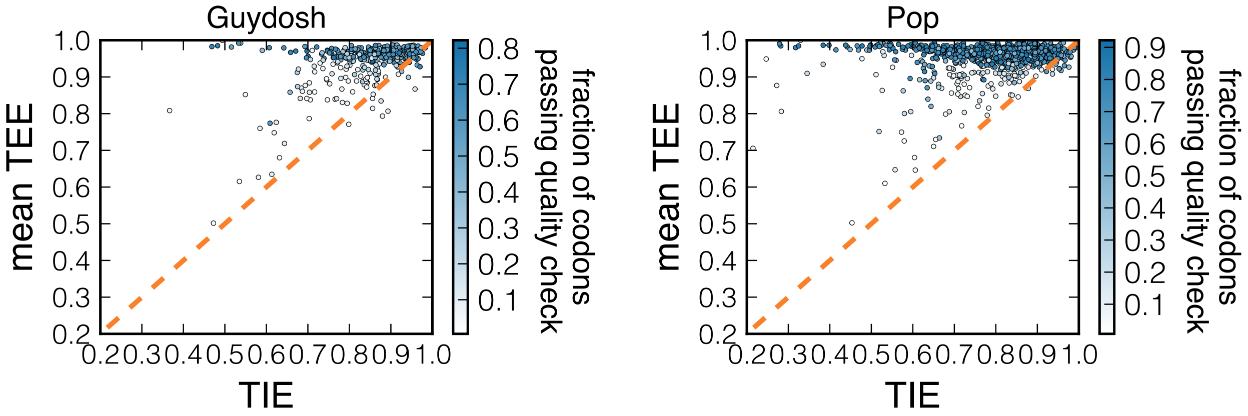

Figure S8. TIE vs mean TEE for the two datasets not shown in the main text.
